# Supplementary figures and images for: An anthranilic acid-responsive transcriptional regulator controls the physiology and pathogenicity of Ralstonia solanacearum
Source: PLoS Pathog. 2022 May 26;18(5):e1010562. doi: 10.1371/journal.ppat.1010562 (PMC9176790; doi:10.1371/journal.ppat.1010562)

*
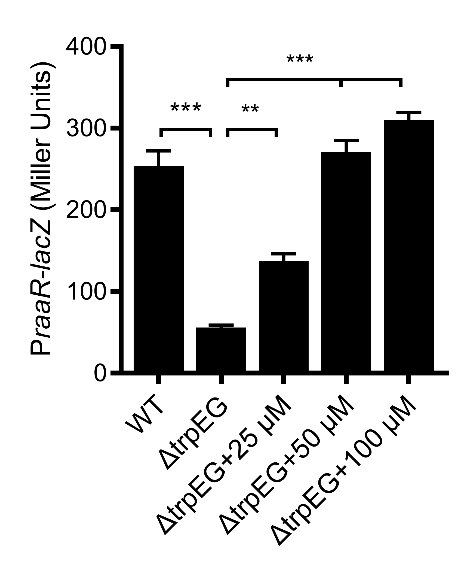
*

Supplement: S1 Fig — The data are the means ± standard deviations of three independent experiments. **p < 0.01; ***p < 0.001 (unpaired t-test). (DOCX) [file ppat.1010562.s001.docx]

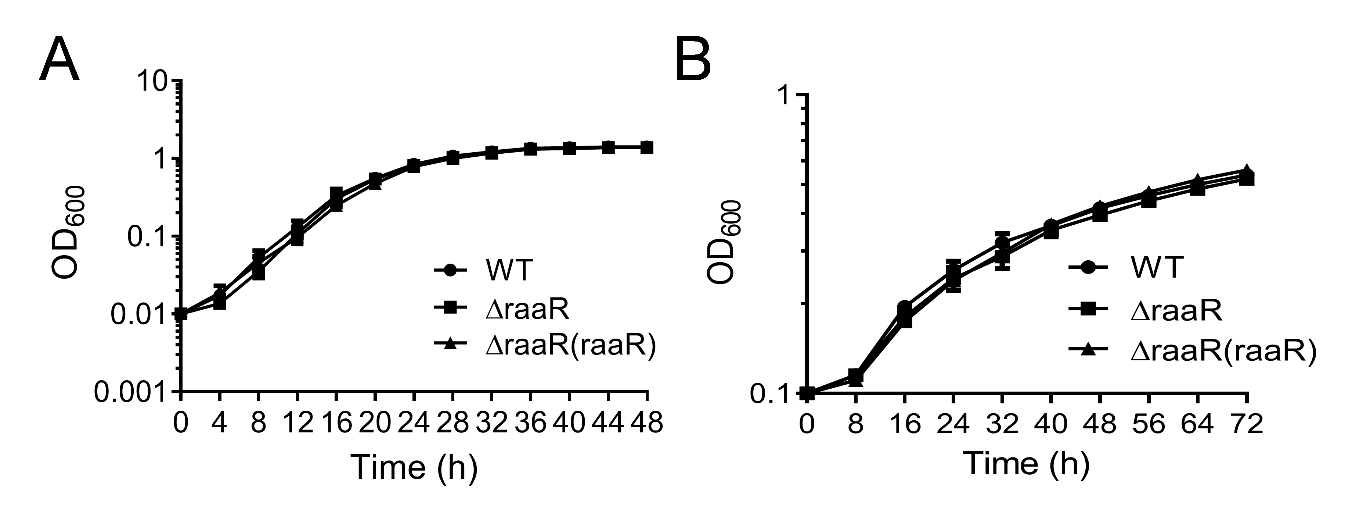

Supplement: S2 Fig — Effects of raaR on the growth curve of R. solanacearum GMI1000 in CPG medium (A) and MP minimal medium (B). The cells were inoculated at 28°C in three replicates with low-intensity shaking in the Bioscreen-C automated growth curve analysis system. The experiment was started at initial OD600 values of 0.01 in CPG medium and 0.1 in MP minimal medium. The data are the means ± standard deviations of three independent experiments. (DOCX) [file ppat.1010562.s002.docx]

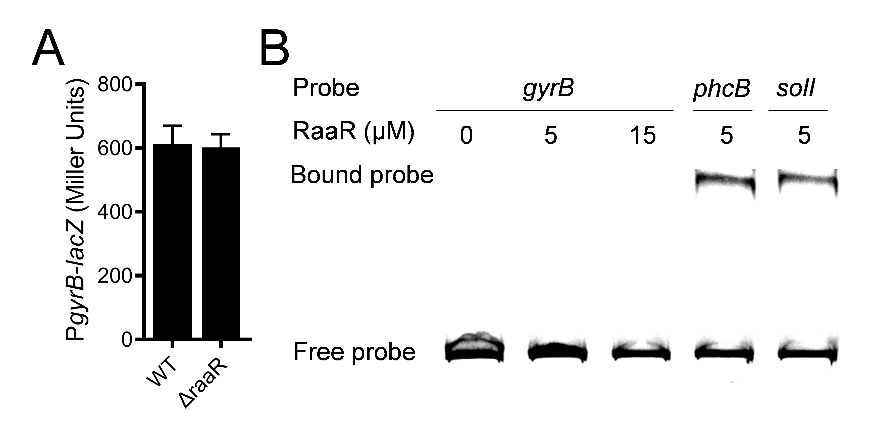

Supplement: S3 Fig — (A) Effect of raaR on the gene expression levels of gyrB in R. solanacearum GMI1000. We used promoter activity assays to quantify gene expression. (B) EMSA analysis of the in vitro binding of RaaR to the promoters of gyrB. (DOCX) [file ppat.1010562.s003.docx]

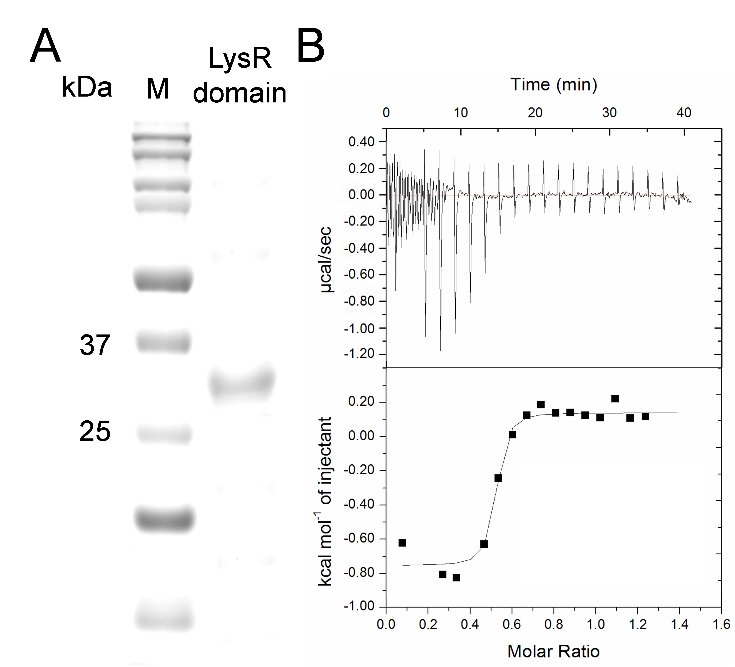

Supplement: S4 Fig — (A) SDS-PAGE of the purified LysR_substrate domain of RaaR protein. (B) ITC titration of 20 μM LysR_substrate domain of RaaR with 250 μM anthranilic acid in PBS buffer at 25°C. (DOCX) [file ppat.1010562.s004.docx]

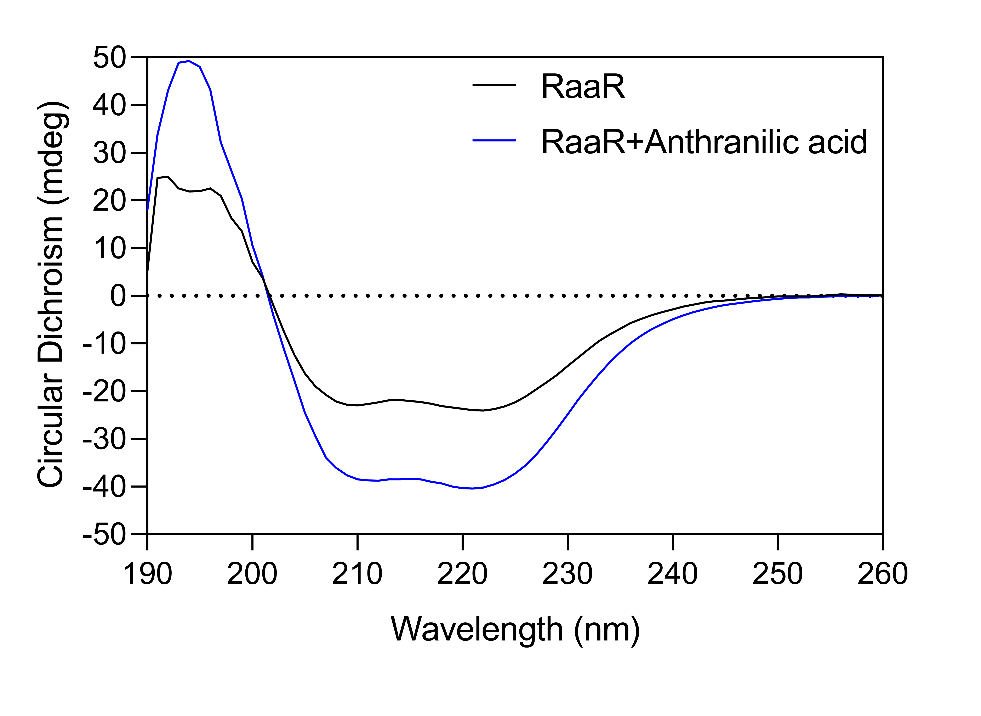

Supplement: S5 Fig — The α-helix and β-sheet spectra of the RaaR protein were changed after RaaR protein was supplemented with anthranilic acid at a final concentration of 10 μM. (DOCX) [file ppat.1010562.s005.docx]

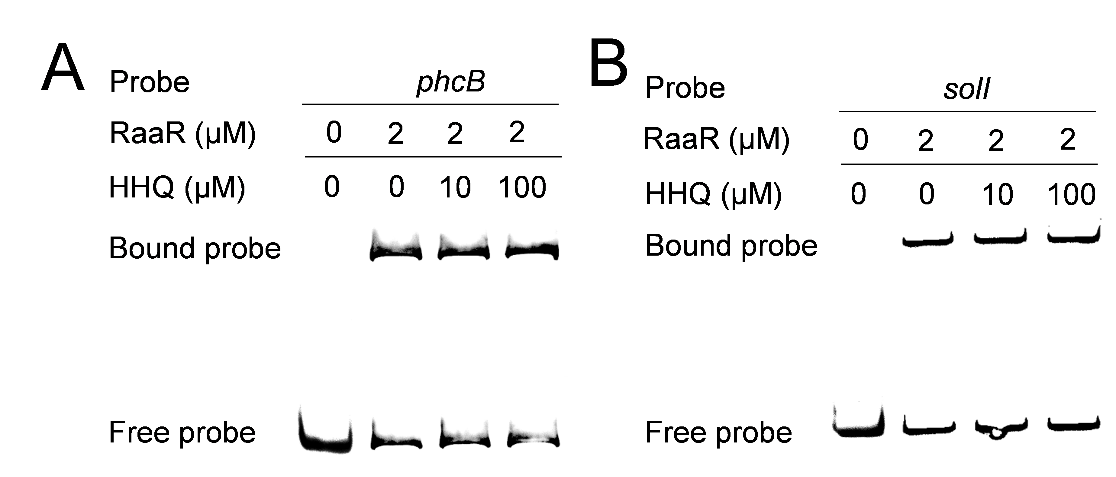

Supplement: S6 Fig — A protein-DNA complex was formed when the protein was incubated with the probes, and different concentrations of HHQ showed no effect on the formation of the complex at room temperature for 30 min. (DOCX) [file ppat.1010562.s006.docx]

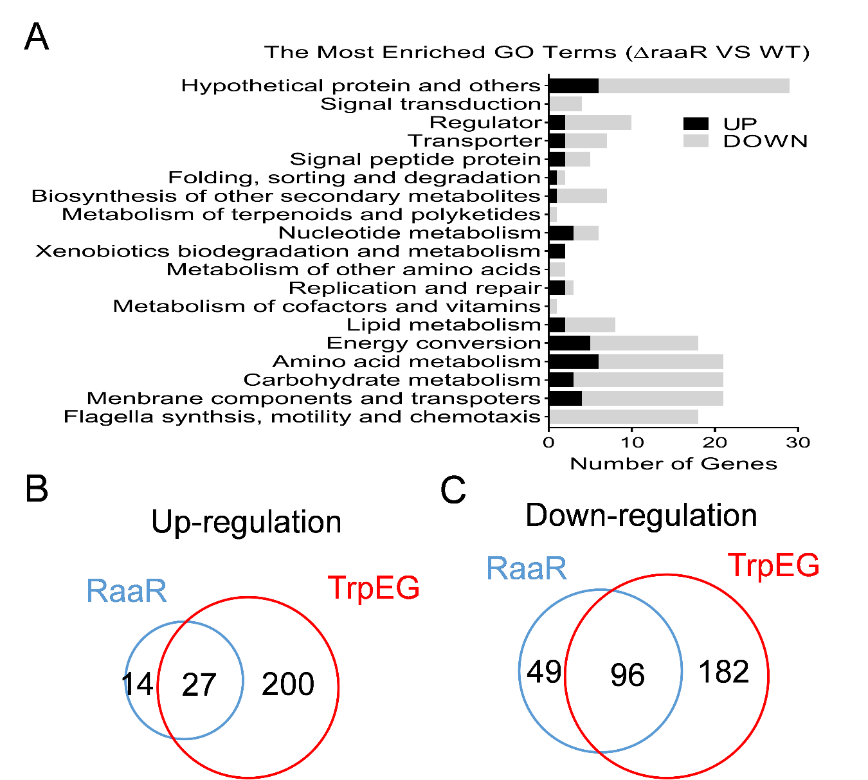

Supplement: S7 Fig — (A) GO term enrichment analysis of differentially expressed genes between the ΔraaR and wild-type strains. Venn diagrams showing the overlap of genes with (B) upregulated or (C) downregulated expression on different mutant backgrounds. Divergently regulated genes are not depicted in these Venn diagrams but are found in S2 Table. (DOCX) [file ppat.1010562.s007.docx]

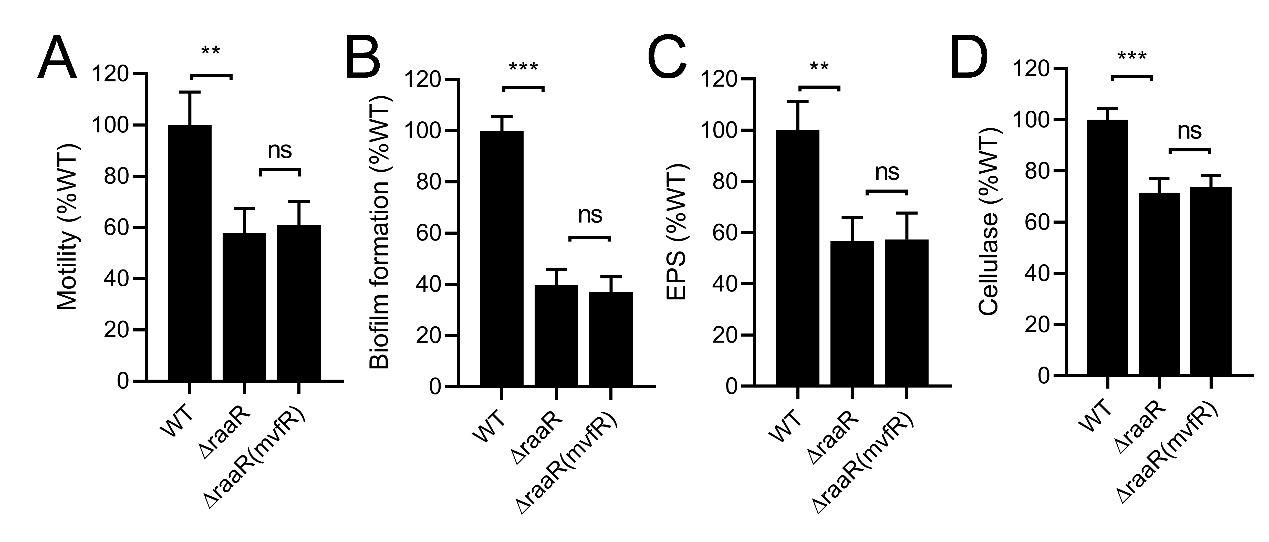

Supplement: S8 Fig — Effects of mvfR of P. aeruginosa on the RaaR-regulated motility (A), biofilm formation (B), EPS production (C) and cellulase production (D) in the R. solanacearum raaR deletion mutant strain. The data are the means ± standard deviations of three independent experiments. **p < 0.01; ***p < 0.001 (unpaired t-test). (DOCX) [file ppat.1010562.s008.docx]

*
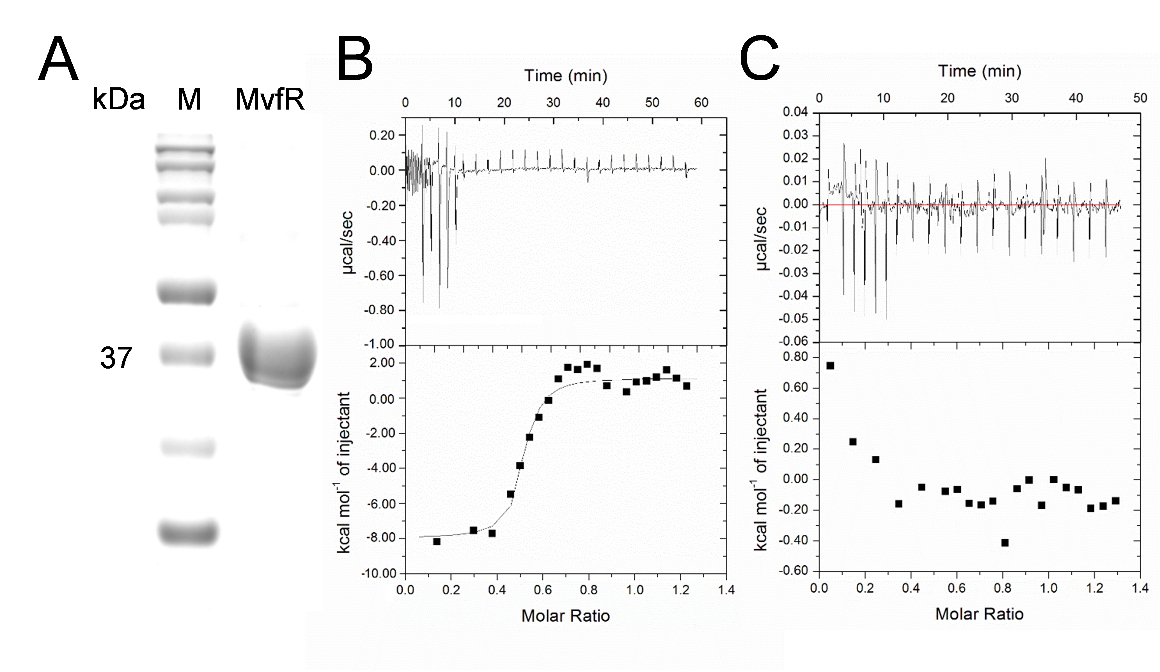
*

Supplement: S9 Fig — (A) SDS-PAGE of the purified MvfR protein. (B) ITC titration of 20 μM MvfR protein with 250 μM PQS in PBS buffer at 25°C. (C) ITC titration of 20 μM MvfR with 250 μΜ anthranilic acid in PBS buffer at 25°C. (DOCX) [file ppat.1010562.s009.docx]

*
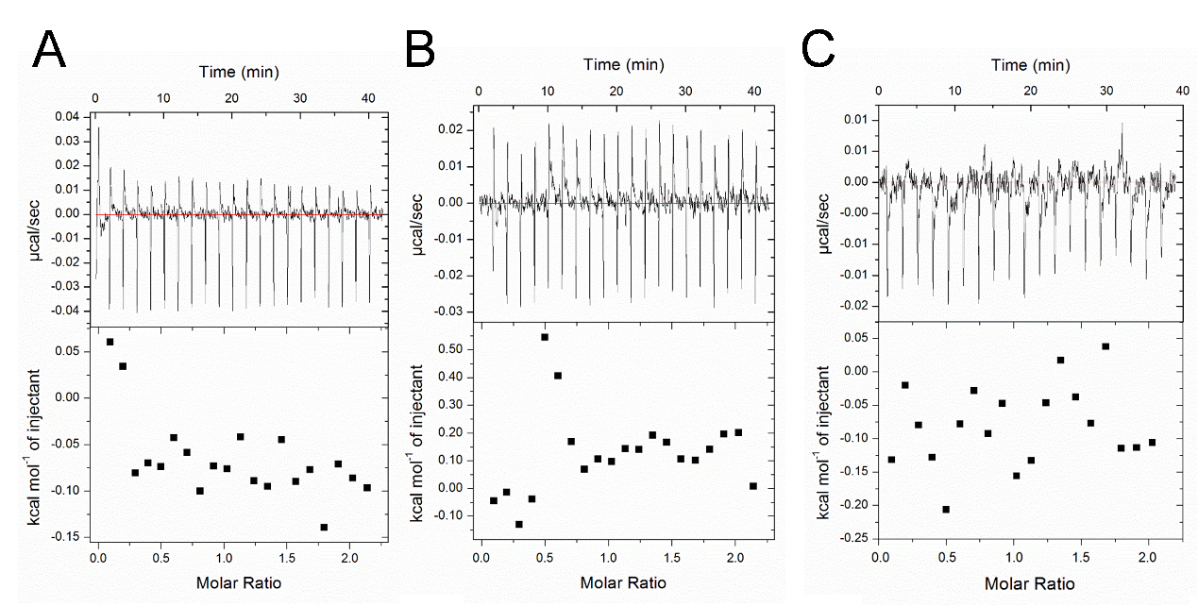
*

Supplement: S10 Fig — ITC analysis of the binding between (A) PQS, (B) HHQ and (C) DHQ and RaaR protein. ITC titration of 20 μM RaaR protein with 250 μM PQS, HHQ and DHQ in PBS buffer at 25°C. (DOCX) [file ppat.1010562.s010.docx]

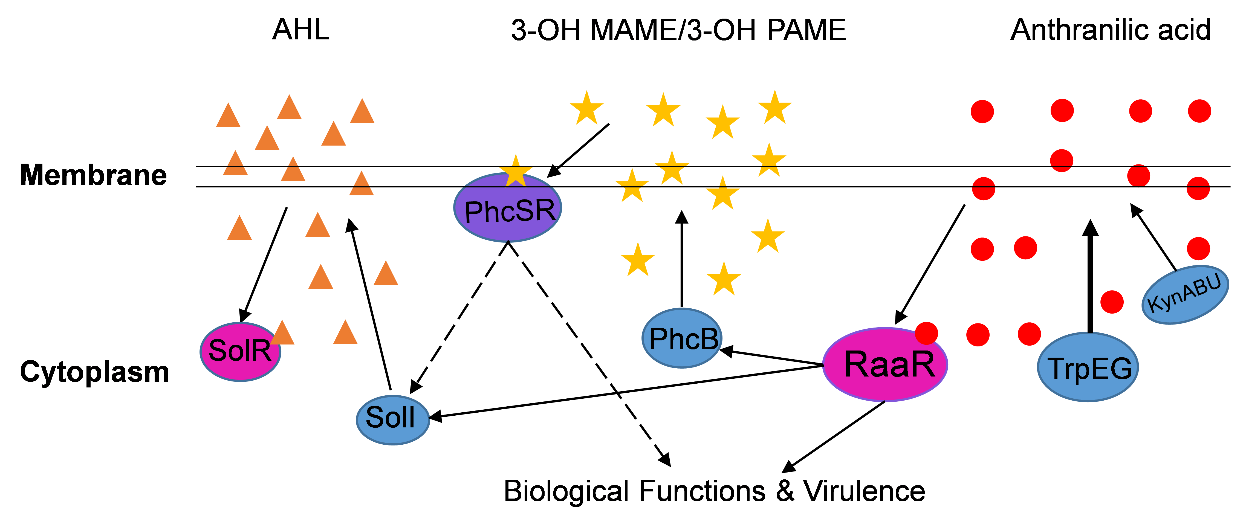

Supplement: S11 Fig — RaaR is involved in sensing anthranilic acid signals and increases the expression levels of PhcB and SolI, which are required for the synthesis of 3-OH PAME/3-OH MAME and AHL signals, respectively. At the same time, the anthranilic acid/RaaR signaling system also directly controls the motility, biofilm formation, and virulence of R. solanacearum. (DOCX) [file ppat.1010562.s011.docx]

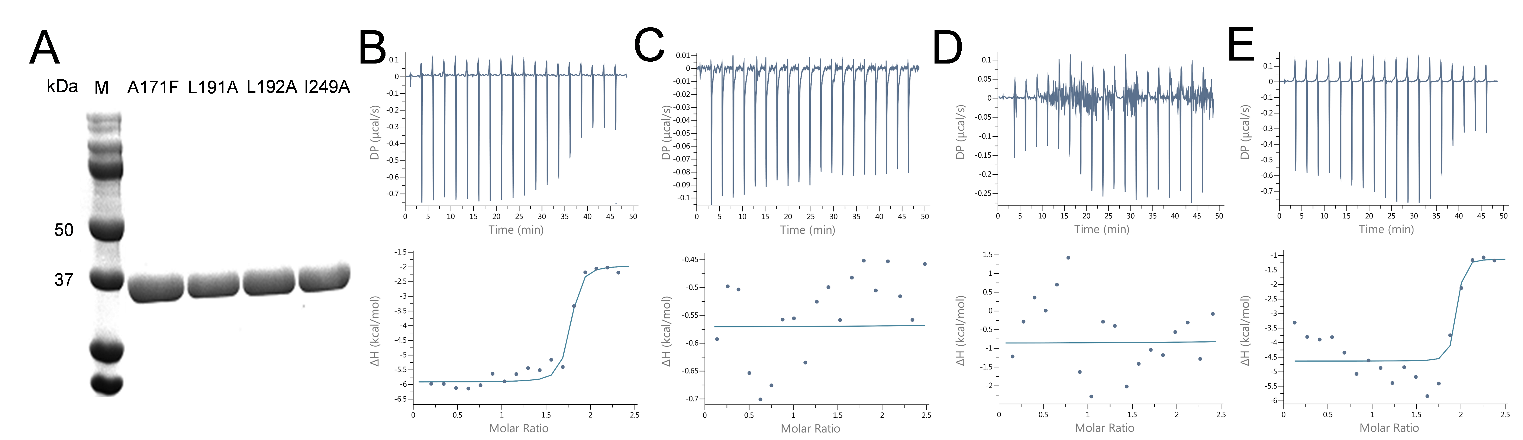

Supplement: S12 Fig — (A) SDS-PAGE of the purified RaaRA171F, RaaRL191A, RaaRL192A and RaaRI249A proteins. ITC analysis of the binding of anthranilic acid to (B) RaaRA171F, (C) RaaRL191A, (D) RaaRL192A and (E) RaaRI249A proteins. (DOCX) [file ppat.1010562.s012.docx]

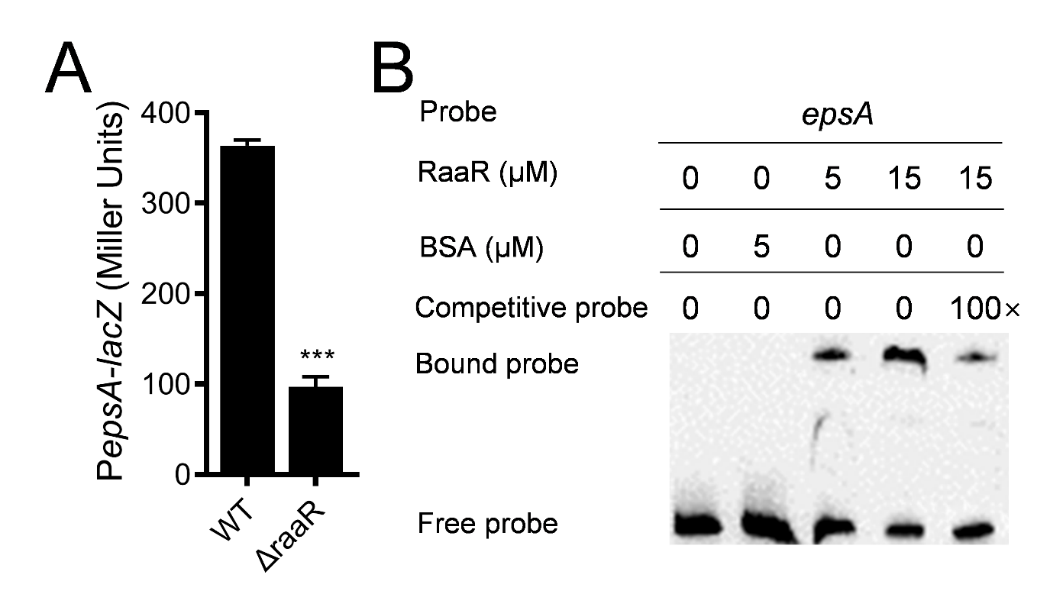

Supplement: S13 Fig — (A) Effects of raaR on the gene expression levels of epsA in R. solanacearum GMI1000. We used promoter activity assays to quantify gene expression. (B) EMSA analysis of the in vitro binding of RaaR to the promoter of epsA. The biotin-labeled 336-bp epsA promoter DNA probe was used for the protein binding assay. A protein-DNA complex, represented by a band shift, was formed when different concentrations of RaaR protein were incubated with the probe at room temperature for 30 min. (DOCX) [file ppat.1010562.s013.docx]

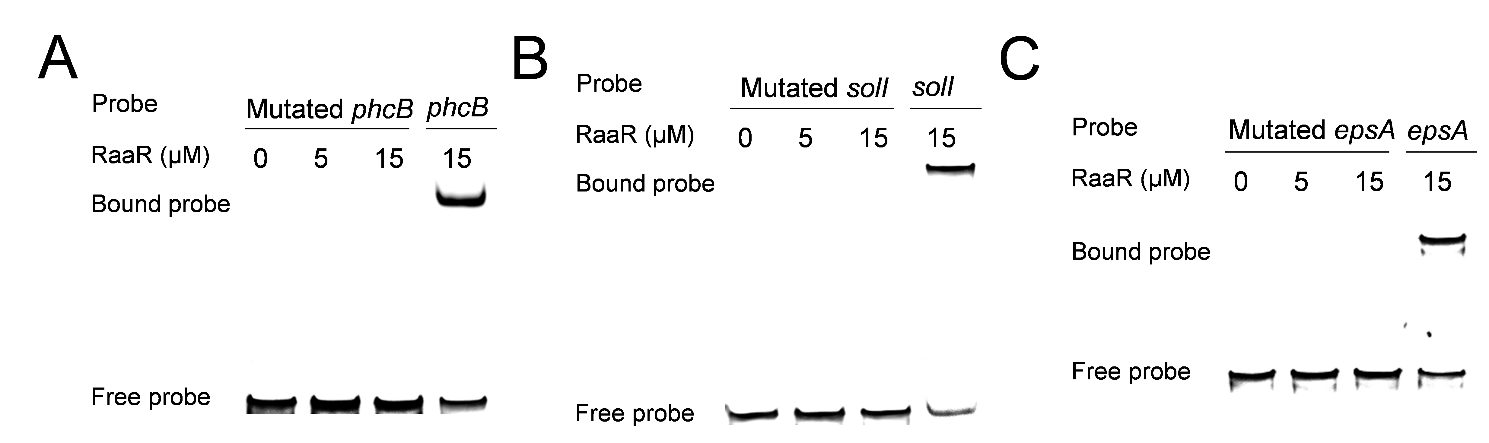

Supplement: S14 Fig — Analysis of the binding between RaaR and the mutated phcB (A), solI (B) and epsA (C) promoters with deletion of the RaaR binding sequence GCGGGTGCG. EMSA analysis was performed in vitro. (DOCX) [file ppat.1010562.s014.docx]
