# Supplementary material for: An anthranilic acid-responsive transcriptional regulator controls the physiology and pathogenicity of Ralstonia solanacearum
Source: PLoS Pathog. 2022 May 26;18(5):e1010562. doi: 10.1371/journal.ppat.1010562 (PMC9176790; doi:10.1371/journal.ppat.1010562)
Supplement: S1 Table — (DOCX) [file ppat.1010562.s015.docx]

**S1** **Table.** Analysis of the homologs of MvfR in *R. solanacearum*

| **Gene ID** | **Gene product** | **MvfR homologue Identity (%)** | **Domain structure analysis** |
| --- | --- | --- | --- |
| *raaR* | probable transcription regulator protein | 30.19 | Fig 1A |
| *RSc1880* | probable transcription regulator protein | 33.5 | 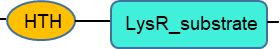 |
| *RSc0542* | probable transcription regulator protein | 16.22 | 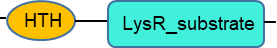 |
| *RSc0615* | probable transcription regulator protein | 17.66 | 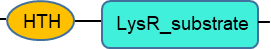 |
| *RSc1110* | probable transcription regulator protein | 16.96 | 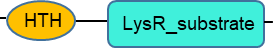 |
| *RSp0942* | probable nitrogen assimilation regulatory transcription regulator protein | 15.18 | 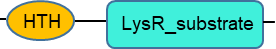 |
| *RSc1472* | probable transcription regulator protein | 17.84 | 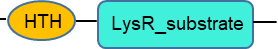 |
| *RSc2537* | probable transcription regulator protein | 18.21 | 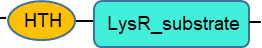 |
| *RSc2761* | probable transcription regulator protein | 18.15 | 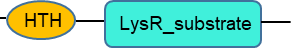 |
| *RSc3332* | probable transcription regulator protein | 16.47 | 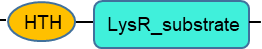 |
