# Supplementary material for: An anthranilic acid-responsive transcriptional regulator controls the physiology and pathogenicity of Ralstonia solanacearum
Source: PLoS Pathog. 2022 May 26;18(5):e1010562. doi: 10.1371/journal.ppat.1010562 (PMC9176790; doi:10.1371/journal.ppat.1010562)
Supplement: S2 Table — Significantly differentially expressed genes were determined by Cufflinks after Benjamini-Hochberg correction. The fold-change is the ratio of the mutant FPKM to the wild-type FPKM. (DOCX) [file ppat.1010562.s016.docx]

**S2** **Table.** List of genes differentially expressed in the *trpEG* and *raaR* mutants compared to the wild-type strain (Log_2_-fold change ≥ 1.5). Significantly differentially expressed genes were determined by Cufflinks after Benjamini-Hochberg correction. The fold-change is the ratio of the mutant FPKM to the wild-type FPKM.

| **Class** | **Gene ID^a^** | **Fold change**   \| ***trpEG*** \| ***raaR*** \| \| --- \| --- \| | | | **Description** |
| --- | --- | --- | --- | --- | --- | --- | --- |
| Flagella synthesis, attachment, motility and chemotaxis | RS_RS05755 | -1.68 | | -2.16 | chemotaxis protein |
|  | RS_RS05760 | -2.65 | | -1.74 | methyl-accepting chemotaxis protein |
|  | RS_RS19040 | -1.75 | | -2.87 | flagellar motor switch protein FliG |
|  | RS_RS23695 | -2.97 | |  | methyl-accepting chemotaxis protein |
|  | RS_RS23885 | -1.58 | | -2.49 | protein phosphatase CheZ |
|  | RS_RS23890 | -1.64 | | -2.07 | response regulator |
|  | RS_RS23895 | -2.90 | |  | chemotaxis response regulator protein-glutamate methylesterase |
|  | RS_RS23900 | -1.91 | |  | probable chemotaxis protein |
|  | RS_RS23905 | -2.52 | | -3.82 | chemotaxis protein CheR |
|  | RS_RS23915 | -2.43 | | -2.51 | chemotaxis protein CheW |
|  | RS_RS23925 | -1.59 | | -2.40 | response regulator |
|  | RS_RS23935 | -1.80 | | -3.65 | flagellar motor protein MotA |
|  | RS_RS18840 | -2.20 | |  | flagellar hook-associated protein FlgK |
|  | RS_RS18845 | -2.45 | | -3.21 | flagellar hook-associated protein 3 |
|  | RS_RS18955 | -2.66 | |  | flagellar export apparatus protein FliQ |
|  | RS_RS18960 | -2.27 | | -2.17 | flagellar biosynthetic protein FliP |
|  | RS_RS23840 | -1.53 | | -3.12 | flagellar biosynthesis regulator FlhF |
|  | RS_RS18995 | -4.10 | | -3.2 | flagellin |
|  | RS_RS19000 | -2.40 | | -2.34 | flagellar hook protein FliD |
|  | RS_RS19005 | -2.02 | |  | flagellar protein FliS |
|  | RS_RS19045 | -1.77 | | -3.58 | flagellar assembly protein FliH |
|  | RS_RS19055 | -1.85 | | -3.28 | flagellar export protein FliJ |
|  | RS_RS23930 |  | | -2.37 | flagellar motor protein MotB |
|  | RS_RS23920 |  | | -2.61 | chemotaxis protein CheA |
| Membrane components and transporters | RS_RS00425 | -2.61 | |  | amino acid ABC transporter substrate-binding protein |
|  | RS_RS04575 | 1.79 | |  | methionine import ATP-binding protein MetN |
|  | RS_RS06700 | 2.41 | |  | ABC transporter permease |
|  | RS_RS06710 | 2.05 | |  | sulfonate ABC transporter substrate-binding protein |
|  | RS_RS21970 |  | | -4.26 | membrane protein |
|  | RS_RS06720 | 2.77 | |  | sulfonate ABC transporter |
|  | RS_RS06725 | 1.91 | |  | aliphatic sulfonate ABC transporter ATP-binding protein |
|  | *cysT* | 3.22 | |  | sulfate ABC transporter permease subunit CysT |
|  | RS_RS06915 | -1.75 | |  | ABC transporter ATP-binding protein |
|  | RS_RS06920 | -2.68 | | -1.74 | glutathione ABC transporter substrate-binding protein |
|  | RS_RS06925 | -2.35 | |  | glutathione ABC transporter permease GsiC |
|  | RS_RS06930 | -1.69 | |  | glutathione ABC transporter permease GsiD |
|  | RS_RS08840 | -2.37 | |  | branched-chain amino acid ABC transporter substrate-binding protein |
|  | RS_RS20950 |  | | -4.23 | anion transporter |
|  | RS_RS11350 | 1.97 | |  | amino acid ABC transporter substrate-binding protein |
|  | RS_RS12235 | -1.73 | |  | ABC transporter ATP-binding protein |
|  | RS_RS12240 | -1.58 | |  | ABC transporter ATP-binding protein |
|  | RS_RS12255 | -1.96 | | -2.04 | branched chain amino acid ABC transporter substrate-binding protein |
|  | *pstS* | 1.93 | |  | phosphate ABC transporter substrate-binding protein PstS |
|  | RS_RS16745 | -1.97 | |  | ABC transporter permease |
|  | RS_RS16970 | 2.20 | |  | metal ABC transporter substrate-binding protein |
|  | RS_RS20175 | -1.51 | | -4.65 | ABC transporter ATP-binding protein |
|  | RS_RS23795 | 2.06 | |  | sulfonate ABC transporter substrate-binding protein |
|  | RS_RS24755 | 4.02 | |  | branched chain amino acid ABC transporter substrate-binding protein |
|  | *xylF* | -1.80 | |  | D-xylose ABC transporter substrate-binding protein |
|  | *gspG* | 1.98 | |  | type II secretion system protein GspG |
|  | RS_RS21245 | -2.44 | |  | EscR/YscR/HrcR family type III secretion system export apparatus protein |
|  | RS_RS15330 | -3.65 | |  | ABC transporter substrate-binding protein |
|  | RS_RS15305 | -2.33 | |  | ABC transporter |
|  | RS_RS15315 | -2.29 | |  | ABC transporter permease |
|  | RS_RS15310 | -2.17 | |  | ABC transporter ATP-binding protein |
|  | RS_RS15320 | -1.98 | |  | sugar ABC transporter permease |
|  | RS_RS21780 | -14.75 | | -10.86 | zinc-binding protein |
|  | RS_RS20855 | -5.24 | | -9.44 | phosphopantetheine-binding and acyl carrier protein |
|  | RS_RS21145 | -2.10 | | -6.64 | peptidoglycan-binding protein LysM |
|  | RS_RS04325 | -1.90 | |  | DNA-binding protein |
|  | RS_RS20575 | -1.72 | | -3.74 | FMN-binding protein |
|  | RS_RS06420 | -12.21 | |  | membrane protein |
|  | RS_RS14870 | -8.05 | |  | membrane protein |
|  | RS_RS22145 |  | | -6.23 | efflux transporter outer membrane subunit |
|  | RS_RS20875 | -4.77 | | -7.99 | membrane protein |
|  | RS_RS06425 | -3.21 | |  | probable transmembrane protein |
|  | RS_RS04355 | -3.93 | |  | probable transmembrane protein |
|  | RS_RS21800 | -3.41 | | -6.25 | probable transmembrane protein |
|  | RS_RS19745 | -2.80 | | -2.26 | probable transmembrane protein |
|  | RS_RS06485 | -2.74 | | -2.79 | outer membrane CHANEL lipoprotein |
|  | RS_RS10875 | -2.35 | |  | membrane protein |
|  | RS_RS15325 | -2.33 | |  | membrane protein |
|  | RS_RS20980 |  | | -3.52 | membrane protein |
|  | RS_RS13650 | -2.15 | |  | membrane protein |
|  | RS_RS18670 | -2.13 | |  | membrane protein |
|  | RS_RS10880 | -2.03 | |  | membrane protein |
|  | RS_RS07450 | -2.31 | |  | probable transmembrane protein |
|  | RS_RS12175 | 2.92 | |  | anion permease |
|  | RS_RS23260 | -3.00 | |  | anion permease |
|  | RS_RS23240 | -1.75 | |  | anion permease |
|  | RS_RS25155 | 1.60 | |  | adenine permease |
|  | RS_RS25270 | -2.56 | |  | probable pseudogene (type III effector protein, avrpm1 homologue) |
|  | RS_RS26630 | -2.33 | |  | type III effector protein (plasmid) |
|  | RS_RS18605 | -1.90 | |  | probable avrpphd family type III effector protein |
|  | RS_RS22065 | 2.52 | |  | Type VI secretion system (T6SS), amidase immunity protein |
|  | RS_RS19630 | 2.34 | |  | mechanosensitive ion channel protein MscS |
|  | RS_RS22180 |  | | -4.21 | APC family permease |
|  | RS_RS10175 | -2.24 | |  | lipoprotein transmembrane |
|  | RS_RS09330 | -2.23 | |  | probable transmembrane protein |
|  | RS_RS00895 | -1.92 | |  | probable transmembrane protein |
|  | RS_RS08970 | -1.87 | |  | membrane protein |
|  | RS_RS00980 | -1.67 | |  | probable transmembrane protein |
|  | RS_RS22975 | -1.58 | |  | lipoprotein transmembran |
|  | RS_RS15950 | -1.56 | |  | probable transmembrane protein |
|  | RS_RS26160 | -1.52 | |  | membrane protein |
|  | RS_RS21855 | 1.50 | | 2.68 | probable transmembrane protein |
|  | RS_RS17695 | 1.55 | |  | membrane protein |
|  | RS_RS12385 | 1.55 | |  | membrane protein |
|  | RS_RS16720 | 1.58 | |  | membrane protein |
|  | RS_RS15880 | 1.91 | |  | ATP-binding protein |
|  | RS_RS17820 | 1.58 | |  | membrane protein |
|  | RS_RS19430 | 1.65 | |  | hypothetical transmembrane protein |
|  | RS_RS24475 | 1.68 | |  | rhomboid family intramembrane serine protease |
|  | RS_RS16495 | 1.69 | | 3.53 | membrane protein |
|  | RS_RS19590 | 1.71 | |  | membrane protein |
|  | RS_RS24995 | 1.79 | |  | membrane protein |
|  | RS_RS25090 | 1.80 | |  | membrane protein |
|  | RS_RS17700 | 1.81 | |  | membrane protein |
|  | RS_RS26205 | 1.87 | |  | probable transporter lipoprotein transmembrane |
|  | RS_RS22075 | 2.10 | | -4.24 | membrane protein |
|  | RS_RS25130 | 2.13 | |  | membrane protein (plasmid) |
|  | RS_RS25095 | 2.25 | |  | membrane protein |
|  | RS_RS22780 | 2.33 | |  | probable transmembrane protein |
|  | RS_RS19105 | 2.50 | |  | probable transmembrane protein |
|  | RS_RS19120 | 2.90 | |  | probable transmembrane protein |
|  | RS_RS21455 | 3.54 | | 2.28 | probable transmembrane protein |
|  | RS_RS17850 | 3.82 | |  | probable transmembrane protein |
|  | RS_RS21465 | 3.97 | | 1.61 | transmembrane protein |
|  | RS_RS09345 | -2.06 | |  | hypothetical transmembrane protein |
| signal peptide protein | RS_RS19095 | -3.64 | |  | signal peptide protein |
|  | RS_RS24185 | -2.78 | |  | probable signal peptide protein |
|  | RS_RS22120 | -1.89 | | -2.36 | probable signal peptide protein |
|  | RS_RS24355 | -1.75 | |  | probable signal peptide protein |
|  | RS_RS23265 | -1.72 | |  | probable signal peptide protein |
|  | RS_RS22755 | -1.72 | |  | probable cog4727, uncharacterized conserved in bacteria signal peptide protein |
|  | RS_RS20455 | -1.56 | | -3.45 | dehydrogenase (Flavoproteins) signal peptide |
|  | RS_RS01680 | -1.52 | |  | probable signal peptide protein |
|  | RS_RS17705 | 1.51 | |  | signal peptide protein |
|  | RS_RS15340 | 1.57 | |  | signal peptide protein |
|  | RS_RS20520 | 1.69 | | -4.18 | probable signal peptide protein |
|  | RS_RS25045 | 1.84 | |  | probable signal peptide protein |
|  | RS_RS14440 | 1.84 | |  | signal peptide protein |
|  | RS_RS23115 | 1.93 | |  | probable signal peptide protein |
|  | RS_RS04630 | 1.94 | |  | probable signal peptide protein |
|  | RS_RS19995 | 2.10 | | 3.58 | probable signal peptide protein |
|  | RS_RS22705 | 2.45 | |  | probable signal peptide protein |
|  | RS_RS16915 | 2.58 | |  | hypothetical signal peptide protein |
|  | RS_RS21470 | 4.72 | | 3.14 | signal peptide protein |
|  | RS_RS25115 | 4.82 | |  | signal peptide protein |
|  | RS_RS09370 | -7.83 | |  | signal peptidase |
|  | RS_RS23970 | -3.00 | |  | peptide synthetase |
|  | *prmC* | 1.51 | |  | probable methylase of polypeptide chain release factors protein |
|  | RS_RS07530 | 1.58 | |  | tetratricopeptide repeat family protein |
| Carbohydrate metabolism | RS_RS10685 | -1.61 | |  | D-arabinitol 4-dehydrogenase |
|  | *galU* | 3.15 | |  | UTP--glucose-1-phosphate uridylyltransferase |
|  | RS_RS21345 | -4.19 | | -2.95 | polygalacturonase |
|  | RS_RS17735 | -1.57 | | -1.82 | 2-methylcitrate synthase |
|  | RS_RS06235 |  | | -2.71 | sugar phosphate isomerase/epimerase |
|  | RS_RS06170 | -1.58 | |  | transaldolase |
|  | RS_RS24610 | -1.65 | |  | 6-phosphogluconolactonase |
|  | RS_RS21745 |  | | -2.16 | L-threonine 3-dehydrogenase |
|  | RS_RS24620 | -1.50 | |  | phosphogluconate dehydratase |
|  | RS_RS10755 | -1.74 | |  | D-tagatose-bisphosphate aldolase, class II, non-catalytic subunit |
|  | RS_RS13785 | -1.67 | |  | gluconolaconase |
|  | RS_RS00960 | -1.87 | |  | alcohol dehydrogenase |
|  | RS_RS15715 | -2.88 | |  | aldehyde dehydrogenase |
|  | RS_RS26195 | -1.69 | |  | glyoxalase i, nickel isomerase |
|  | RS_RS13685 | -1.53 | | -3.17 | SAM-dependent methyltransferase PhcB |
|  | RS_RS13750 | -1.50 | | -2.46 | transcriptional regulator PhcA |
|  | RS_RS22015 | -1.57 | | -2.28 | EPS I polysaccharide export inner membrane protein EpsF |
|  | RS_RS22020 | -2.38 | | -4.08 | EPS I polysaccharide export inner membrane protein EpsE |
|  | RS_RS22025 | -1.72 | | -4.72 | UDP-N-acetyl-D-mannosamine dehydrogenase |
|  | RS_RS22030 | -1.65 | | -2.22 | UDP-N-acetylglucosamine 2-epimerase (non-hydrolyzing) |
|  | RS_RS22035 | -1.53 | | -2.89 | tyrosine protein kinase |
|  | RS_RS22045 | -1.79 | | -2.61 | EPS I polysaccharide export outer membrane protein EpsA |
|  | RS_RS18695 | 1.72 | |  | 2-isopropylmalate synthase 2 |
|  | RS_RS26155 |  | | -1.53 | glycoside hydrolase family 6 protein |
|  | RS_RS16005 | 3.69 | |  | glycoside hydrolase |
|  | RS_RS22310 | 2.14 | |  | glucose-fructose oxidoreductase |
|  | RS_RS20890 | -5.03 | | -6.18 | alpha/beta hydrolase |
|  | RS_RS20840 | -3.71 | | -5.88 | alpha/beta hydrolase |
|  | RS_RS23965 | -3.49 | |  | alpha/beta hydrolase |
|  | RS_RS22480 | -3.09 | |  | alpha/beta hydrolase |
|  | RS_RS25245 | -1.69 | |  | hydrolase |
|  | RS_RS08950 | -1.50 | |  | alpha/beta hydrolase |
|  | RS_RS20445 | -2.09 | |  | lactoylglutathione lyase |
|  | RS_RS06385 | -4.59 | |  | copper-translocating P-type ATPase |
|  | RS_RS17915 | -1.52 | | -1.94 | glycoside hydrolase family 5 protein |
|  | RS_RS18285 | 1.65 | |  | malto-oligosyltrehalose synthase |
|  | RS_RS18295 | 1.82 | |  | malto-oligosyltrehalose trehalohydrolase |
|  | RS_RS07540 | 1.89 | |  | 2,5-diketo-D-gluconate reductase A |
|  | RS_RS03570 | 2.29 | |  | lytic transglycosylase |
|  | RS_RS18305 | 1.72 | |  | glycogen-branching enzyme |
|  | RS_RS21965 |  | | -1.69 | polysaccharide biosynthesis protein |
|  | RS_RS18310 | 1.67 | |  | alpha-amylase |
|  | RS_RS18315 | 1.82 | |  | alpha-amylase |
|  | RS_RS18320 | 2.91 | |  | glycogen synthase |
|  | RS_RS02545 | 1.69 | |  | mannose-1-phosphate guanylyltransferase |
|  | RS_RS22445 | 1.65 | |  | trehalose-phosphatase |
|  | RS_RS22700 | 2.27 | |  | glycoprotein (plasmid) |
|  | RS_RS21060 |  | | -3.53 | SMP-30/gluconolactonase/LRE family protein |
|  | RS_RS20620 | 2.16 | | 2.52 | trehalose-6-phosphate synthase |
|  | RS_RS24605 | -1.6 | |  | glucokinase |
|  | RS_RS24030 |  | | 1.63 | cupin domain-containing protein |
|  | RS_RS19200 |  | | 2.87 | aldolase |
| Amino acid metabolism | *glyA* | 3.62 | |  | serine hydroxymethyltransferase |
|  | RS_RS26005 | -3.16 | |  | Serine/threonine protein kinase |
|  | RS_RS06935 | -2.24 | |  | D-aminopeptidase |
|  | RS_RS20550 |  | | -5.23 | D-amino-acid transaminase |
|  | RS_RS11355 | 2.30 | |  | D-amino acid dehydrogenase small subunit |
|  | RS_RS14445 |  | | -1.81 | anthranilate phosphoribosyltransferase |
|  | RS_RS16810 | -2.11 | |  | phenylalanine 4-monooxygenase |
|  | RS_RS20005 | -2.63 | | -3.27 | phenylacetate-CoA oxygenase subunit PaaA |
|  | RS_RS20010 | -2.52 | | -3.05 | phenylacetate-CoA oxygenase subunit PaaB |
|  | *paaI* | -1.96 | | -2.17 | phenylacetic acid degradation protein |
|  | *paaJ* | -2.80 | | -2.81 | phenylacetate-CoA oxygenase subunit PaaJ |
|  | RS_RS20910 |  | | -3.73 | pyridoxal phosphate-dependent aminotransferase |
|  | RS_RS20025 | -2.64 | | -4.31 | phenylacetic acid degradation protein |
|  | RS_RS21795 | -7.22 | | -9.23 | peptidase (collagenase-like) |
|  | RS_RS21790 | -6.35 | | -8.72 | U32 family peptidase |
|  | RS_RS04965 | -3.17 | | -3.18 | peptidase S1 |
|  | RS_RS12975 | -2.08 | |  | D-alanyl-D-alanine endopeptidase |
|  | RS_RS15405 | 1.50 | |  | peptidase |
|  | RS_RS14430 |  | | -1.93 | anthranilate synthase component I |
|  | RS_RS14180 | 1.88 | |  | peptidase M23 |
|  | RS_RS16925 | 2.80 | |  | phosphatidylserine synthase |
|  | RS_RS21400 | 2.21 | |  | 2-keto-4-pentenoate hydratase |
|  | *hppD* | 1.56 | |  | 4-hydroxyphenylpyruvate dioxygenase |
|  | RS_RS21395 | 2.06 | | 1.88 | 2-hydroxymuconic semialdehyde dehydrogenase |
|  | RS_RS24725 | -2.41 | |  | catalase |
|  | RS_RS23700 | -3.24 | |  | probable 2-oxoglutarate dehydrogenase e1 decarboxylase component oxidoreductase protein |
|  | RS_RS20380 |  | | -1.78 | indole-3-glycerol phosphate synthase TrpC |
|  | RS_RS02405 | -1.84 | |  | class II glutamine amidotransferase |
|  | RS_RS13240 | -2.71 | |  | imidazolonepropionase |
|  | RS_RS13245 | -2.97 | |  | formimidoylglutamase |
|  | RS_RS13250 | -3.16 | |  | histidine ammonia-lyase |
|  | RS_RS14435 |  | | -2.01 | aminodeoxychorismate/anthranilate synthase component II |
|  | RS_RS13255 | -2.72 | |  | urocanate hydratase |
|  | RS_RS00780 | 1.61 | |  | arginase |
|  | RS_RS03345 | 1.50 | |  | 4-hydroxy-tetrahydrodipicolinate synthase |
|  | *putA* | 1.77 | |  | trifunctional transcriptional regulator/proline dehydrogenase/L-glutamate gamma-semialdehyde dehydrogenase |
|  | RS_RS19480 | 1.67 | |  | ornithine cyclodeaminase |
|  | RS_RS23450 | 2.33 | |  | polyamine aminopropyltransferase 1 |
|  | RS_RS23580 | 2.33 | |  | polyamine aminopropyltransferase 1 |
|  | RS_RS24040 |  | | 2.45 | amino acid adenylation domain-containing protein |
|  | RS_RS00470 | 1.67 | | 1.62 | adenosylhomocysteinase |
|  | RS_RS16465 | -1.71 | | -1.68 | acyl-homoserine-lactone synthase SolI |
|  | RS_RS16905 | 1.58 | |  | 5'-methylthioadenosine/S-adenosylhomocysteine nucleosidase |
|  | RS_RS20210 | 1.52 | | 4.17 | aminocyclopropane-1-carboxylate deaminase/D-cysteine desulfhydrase family protein |
|  | RS_RS20360 | 3.51 | | 3.35 | 5-methyltetrahydropteroyltriglutamate--homocysteine methyltransferase |
|  | RS_RS00145 | -3.49 | |  | 4-aminobutyrate transaminase |
|  | RS_RS19170 |  | | 1.84 | 2,3-diaminopropionate biosynthesis protein SbnA |
|  | RS_RS16525 | 1.98 | |  | glutamate--cysteine ligase |
| Energy conversion | RS_RS06705 | 2.25 | |  | FMN reductase (NADPH) |
|  | RS_RS18940 | 3.65 | |  | 1-acyl-sn-glycerol-3-phosphate acyltransferase |
|  | RS_RS24495 | 2.37 | |  | 2-oxoglutarate-dependent ethylene/succinate-forming enzyme |
|  | RS_RS21495 |  | | -3.24 | FAD-dependent monooxygenase |
|  | RS_RS20490 |  | | -2.17 | FAD-dependent oxidoreductase |
|  | RS_RS06025 | -1.94 | |  | NADPH:quinone oxidoreductase |
|  | RS_RS12160 | 1.76 | |  | phosphoadenosine phosphosulfate reductase |
|  | RS_RS12170 | 2.06 | |  | sulfite reductase |
|  | *cysC* | 2.07 | |  | adenylyl-sulfate kinase |
|  | RS_RS12160 | 1.76 | |  | phosphoadenosine phosphosulfate reductase |
|  | RS_RS20845 | -4.85 | | -6.44 | cystathionine gamma-synthase |
|  | RS_RS09500 | 1.63 | |  | FAD-dependent oxidoreductase |
|  | RS_RS24065 | 1.58 | | 3.92 | probable serine acetyltransferase protein |
|  | RS_RS24745 | 3.58 | |  | FAD-dependent oxidoreductase |
|  | RS_RS00560 | -5.13 | |  | 2-nitropropane dioxygenase |
|  | RS_RS06020 |  | | -1.94 | alkene reductase |
|  | RS_RS18665 | -2.22 | |  | 2-nitropropane dioxygenase |
|  | RS_RS21805 | -3.52 | | -5.78 | MFS transporter |
|  | RS_RS21810 | -3.77 | | -6.71 | Nitrate/nitrite transporter (plasmid) |
|  | RS_RS21815 | -5.54 | | -7.3 | nitrate reductase subunit alpha |
|  | RS_RS21820 | -2.00 | | -4.88 | nitrate reductase 2 (NRZ), beta subunit |
|  | RS_RS23720 | -2.50 | |  | TAT-dependent nitrous-oxide reductase |
|  | RS_RS24360 | -5.31 | |  | probable major anaerobically induced outer membrane transmembrane protein |
|  | RS_RS06395 | -7.85 | |  | cytochrome c oxidase, cbb3-type subunit I |
|  | RS_RS21460 | 3.40 | | 3.51 | dehydrogenase oxidoreductase |
|  | RS_RS06400 | -7.31 | |  | peptidase S41 |
|  | RS_RS06405 | -13.23 | |  | cytochrome oxidase |
|  | RS_RS06410 | -5.10 | |  | probable cytochrome c oxidase (subunit III) transmembrane protein |
|  | RS_RS24225 |  | | -1.63 | aldo/keto reductase |
|  | RS_RS06390 | -13.06 | |  | cytochrome oxidase maturation protein Cbb3 |
|  | RS_RS00985 | -2.77 | |  | cytochrome b |
|  | RS_RS23715 | -2.43 | | -2.07 | Cytochrome c-555 (plasmid) |
|  | RS_RS06415 | -2.22 | |  | cytochrome c oxidase accessory protein CcoG |
|  | RS_RS01065 | -2.15 | | -3.18 | short-chain dehydrogenase |
|  | RS_RS20465 | -1.77 | |  | alcohol dehydrogenase |
|  | RS_RS10600 | -1.77 | |  | oxidoreductase |
|  | RS_RS09225 | -1.60 | |  | Indolepyruvate oxidoreductase subunit IorA |
|  | RS_RS19235 | -1.56 | | -2.60 | oxidoreductase |
|  | RS_RS12165 | 1.50 | |  | oxidoreductase |
|  | RS_RS21480 | 1.57 | | 2.30 | LLM class oxidoreductase |
|  | RS_RS23985 |  | | 1.76 | NAD(P)/FAD-dependent oxidoreductase |
|  | RS_RS23455 | 1.70 | |  | amine oxidase |
|  | RS_RS23585 | 1.70 | |  | amine oxidase |
|  | RS_RS24655 | 1.98 | |  | bb3-type cytochrome oxidase subunit IV |
|  | RS_RS17730 |  | | 2.19 | Fe/S-dependent 2-methylisocitrate dehydratase AcnD |
|  | RS_RS20850 | -4.59 | | -3.97 | aminoacyl-tRNA synthet |
| Lipid metabolism | RS_RS15300 | -1.55 | |  | glycerol-3-phosphate dehydrogenase |
|  | RS_RS20870 | -4.41 | | -4.81 | beta-ketoacyl-ACP synthase |
|  | RS_RS20415 |  | | -5.47 | acyl-CoA dehydrogenase family protein |
|  | RS_RS20900 | -4.51 | | -5.37 | acyl-CoA desaturase |
|  | RS_RS15335 | -1.58 | |  | glycerol kinase |
|  | RS_RS08885 | -1.86 | |  | 3-hydroxyacyl-CoA dehydrogenase |
|  | RS_RS15725 | -1.86 | |  | zinc-dependent alcohol dehydrogenase |
|  | RS_RS20240 |  | | -1.75 | acyl-CoA dehydrogenase |
|  | RS_RS19230 | -2.23 | | -2.27 | alcohol dehydrogenase |
|  | RS_RS24750 | 3.36 | |  | aldehyde dehydrogenase family protein |
|  | RS_RS08835 | -2.35 | |  | long-chain-fatty-acid--CoA ligase |
|  | RS_RS03920 | -1.61 | |  | long-chain-fatty-acid--CoA ligase |
|  | RS_RS14390 |  | | 1.58 | 2-(1,2-epoxy-1,2-dihydrophenyl)acetyl-CoA isomerase |
|  | RS_RS20860 | -5.27 | | -6.51 | omega-3 fatty acid desaturase |
|  | RS_RS12705 | -3.00 | |  | 3-hydroxyacyl-CoA dehydrogenase |
|  | RS_RS06850 | -2.88 | |  | acyl-CoA-binding protein |
|  | RS_RS03155 | 1.81 | |  | lipoprotein |
|  | RS_RS14385 |  | | 1.84 | enoyl-CoA hydratase |
| Transporter | RS_RS02740 | -3.28 | |  | MFS transporter |
|  | RS_RS23960 | -3.24 | |  | MFS transporter |
|  | RS_RS20595 | -2.49 | | -3.93 | MFS transporter |
|  | RS_RS09365 | -2.21 | |  | multidrug transporter |
|  | RS_RS22185 |  | | -5.41 | OFA family MFS transporter |
|  | RS_RS24390 | -1.92 | |  | nitrate ABC transporter substrate-binding protein |
|  | RS_RS10170 | -1.86 | |  | amino acid transporter |
|  | RS_RS00920 | -1.75 | |  | transporter |
|  | RS_RS19740 | -1.71 | | -3.25 | MFS transporter |
|  | RS_RS22495 | -1.68 | |  | RND transporter |
|  | RS_RS22490 | -1.66 | |  | multidrug efflux RND transporter permease subunit |
|  | RS_RS22485 | -1.66 | |  | MexE family multidrug efflux RND transporter periplasmic adaptor subunit |
|  | RS_RS24385 | -1.63 | |  | ABC transporter permease |
|  | RS_RS24380 | -1.61 | |  | sulfonate ABC transporter ATP-binding lipoprotein |
|  | RS_RS20040 |  | | -2.18 | MFS transporter |
|  | RS_RS10870 | -1.56 | |  | C4-dicarboxylate ABC transporter |
|  | RS_RS08865 | -2.57 | |  | transposase |
|  | RS_RS26545 | 1.53 | |  | remnant of isrso16-transposase orfb protein |
|  | RS_RS25305 | 1.79 | |  | probable remnant of a transposase protein |
|  | RS_RS26600 | 1.88 | |  | IS3 family transposase ISRso11 |
|  | RS_RS26380 | 2.37 | |  | transposase |
|  | RS_RS26415 | 2.37 | |  | transposase |
|  | RS_RS15955 | -1.70 | |  | GNAT family N-acetyltransferase |
|  | RS_RS21640 | 1.96 | |  | methyltransferase |
|  | RS_RS14345 | 1.50 | |  | ABC transporter permease |
|  | RS_RS16050 | 1.50 | |  | transporter |
|  | RS_RS17230 | 1.52 | |  | MFS transporter |
|  | RS_RS22405 | 1.55 | |  | peptide transporter |
|  | RS_RS17565 | 1.56 | |  | MFS transporter |
|  | RS_RS20325 | 1.68 | | 3.58 | RND transporter |
|  | RS_RS17330 | -1.55 | |  | DNA-binding protein |
|  | RS_RS11650 | 1.85 | |  | MFS transporter |
|  | RS_RS23525 | 1.87 | |  | RhtB family transporter |
|  | RS_RS23655 | 1.88 | |  | RhtB family transporter |
|  | RS_RS16530 | 2.23 | |  | potassium transporter Kef |
|  | RS_RS17320 | 2.97 | |  | manganese transporter |
|  | RS_RS19560 | 3.32 | |  | MFS transporter |
|  | RS_RS23200 |  | | 1.83 | MFS transporter |
|  | RS_RS01005 | -3.71 | |  | hypothetical h+-transporting two-sector atpase, gamma subunit; protein |
|  | RS_RS20305 | 1.74 | | -3.05 | magnesium and cobalt transport protein CorA |
| Regulator | RS_RS21775 | -3.57 | | -5.97 | Crp/Fnr family transcriptional regulator |
|  | RS_RS05515 | -3.35 | |  | LysR family transcriptional regulator |
|  | RS_RS20410 |  | | -4.17 | AraC family transcriptional regulator |
|  | RS_RS02690 | -3.01 | |  | transcription regulator protein |
|  | RS_RS06490 | -2.70 | | -3.58 | MarR family transcriptional regulator |
|  | RS_RS02340 | -2.35 | |  | TetR family transcriptional regulator |
|  | RS_RS14005 | -2.29 | |  | AsnC family transcriptional regulator |
|  | RS_RS12710 | -1.93 | |  | probable transcription regulator protein |
|  | RS_RS21015 |  | | -3.12 | TetR/AcrR family transcriptional regulator |
|  | RS_RS13815 | -1.90 | |  | LysR family transcriptional regulator |
|  | RS_RS22475 | -1.84 | |  | LysR family transcriptional regulator |
|  | RS_RS05425 | -1.80 | |  | LysR family transcriptional regulator |
|  | RS_RS10035 | -1.76 | |  | probable transcription regulator protein |
|  | RS_RS23130 | -1.73 | |  | transcriptional regulator |
|  | RS_RS03815 | -1.70 | |  | IclR family transcriptional regulator |
|  | RS_RS09350 | -1.67 | |  | MarR family transcriptional regulator |
|  | RS_RS06905 | -1.63 | |  | MurR/RpiR family transcriptional regulator |
|  | RS_RS21860 |  | | -2.12 | helix-turn-helix transcriptional regulator |
|  | RS_RS16695 | -1.58 | |  | LysR family transcriptional regulator |
|  | RS_RS00055 | -1.57 | |  | TetR family transcriptional regulator |
|  | RS_RS21630 | -1.56 | | -2.75 | LysR family transcriptional regulator |
|  | RS_RS20290 |  | | -1.59 | LysR family transcriptional regulator |
|  | RS_RS21740 | -1.55 | | -3.33 | nitric oxide reductase transcription regulator |
|  | RS_RS19830 | -1.50 | |  | transcriptional regulator |
|  | RS_RS01430 | 1.50 | |  | response regulator |
|  | RS_RS25105 | 1.54 | |  | sigma-54-dependent Fis family transcriptional regulator |
|  | RS_RS02765 | 1.57 | |  | regulatory protein RecX |
|  | RS_RS22565 | 1.58 | |  | transcriptional regulator |
|  | RS_RS05345 | 1.59 | |  | probable two-component system response regulator transcription regulator protein |
|  | RS_RS15295 |  | | 1.56 | DeoR/GlpR transcriptional regulator |
|  | RS_RS22745 | 1.68 | |  | transcription regulator protein |
|  | RS_RS25110 | 1.82 | |  | sigma-54-dependent Fis family transcriptional regulator |
|  | RS_RS15820 | 1.98 | |  | transcriptional regulator |
|  | RS_RS23510 | 1.99 | |  | transcriptional regulator |
|  | RS_RS23640 | 1.99 | |  | transcriptional regulator |
|  | RS_RS15395 |  | | 1.89 | response regulator transcription factor |
|  | RS_RS05945 | 2.05 | |  | Fis family transcriptional regulator |
|  | RS_RS15885 | 2.18 | |  | DNA-binding response regulator |
|  | RS_RS09495 | 2.26 | |  | LysR family transcriptional regulator |
|  | RS_RS08435 | 2.60 | |  | Phage regulatory protein CII (CP76) |
|  | RS_RS25480 | 2.64 | |  | prophage regulatory protein |
| Signal transduction | RS_RS21840 | -5.57 | | -6.23 | histidine kinase |
|  | RS_RS21845 | -2.94 | | -3.08 | DNA-binding response regulator |
|  | RS_RS26305 | 1.89 | |  | Sensor signal transduction histidine kinase |
|  | RS_RS18210 | 2.66 | |  | two-component sensor histidine kinase |
|  | RS_RS20255 |  | | -1.79 | heavy metal response regulator transcription factor |
|  | RS_RS04725 | 1.55 | |  | L-histidine N(alpha)-methyltransferase |
|  | RS_RS17955 | 1.74 | |  | protein prenyltransferase |
|  | RS_RS20880 | -4.81 | | -3.65 | carbamoyltransferase |
|  | RS_RS19025 | -1.56 | |  | mannosyltransferase |
|  | RS_RS00905 | -4.52 | |  | histidine kinase |
|  | RS_RS25120 | 2.37 | |  | RNA polymerase sigma-54 factor |
|  | RS_RS13655 | -2.24 | |  | TonB-dependent siderophore receptor |
| Metabolism of cofactors and vitamins | RS_RS16455 | -7.36 | |  | coproporphyrinogen III oxidase |
|  | RS_RS09190 | 2.78 | |  | probable maleate cis-trans isomerase protein |
|  | RS_RS00555 | 1.60 | |  | phosphomethylpyrimidine synthase |
|  | RS_RS17975 | 2.24 | | -2.52 | NIFS-like protein |
| Replication and repair | RS_RS02760 | 1.80 | |  | DNA recombination/repair protein RecA |
|  | RS_RS18345 | 2.30 | | 2.24 | transcriptional repressor |
|  | RS_RS20930 | 2.22 | | 4.44 | cell division protein |
|  | RS_RS01575 | -6.16 | | -1.85 | twin-arginine translocation protein |
| Metabolism of other amino acids | RS_RS10110 | -1.85 | |  | glutathione S-transferase |
|  | RS_RS16580 | 2.14 | |  | glutathione S-transferase |
|  | RS_RS18475 | -2.46 | |  | gamma-glutamyltransferase |
|  | RS_RS00415 | -1.80 | |  | aspartate aminotransferase family protein |
|  | RS_RS06940 | -1.77 | |  | D-aminopeptidase |
|  | RS_RS19110 | 2.66 | |  | peptidase |
|  | RS_RS21065 | -1.73 | | -2.77 | spermidine synthase |
|  | RS_RS19555 | 2.74 | |  | methionine biosynthesis protein MetW |
|  | RS_RS19485 | 2.14 | |  | amidinotransferase |
|  | RS_RS00420 | -2.07 |  | | 3-keto-5-aminohexanoate cleavage enzyme |
|  | RS_RS07510 | -2.29 | -3.13 | | Valine--pyruvate transaminase |
| Xenobiotics biodegradation and metabolism | RS_RS20525 | 2.04 | 5.28 | | cytochrome P450 |
|  | RS_RS08945 | -2.69 |  | | 4-hydroxybutyrate dehydrogenase |
|  | RS_RS23460 | 1.86 |  | | carboxymethylenebutenolidase |
|  | RS_RS10890 | -1.86 |  | | allophanate hydrolase |
|  | RS_RS23590 | 1.86 |  | | carboxymethylenebutenolidase |
|  | RS_RS09535 | 1.62 |  | | alkylhydroperoxidase |
|  | RS_RS02215 | 2.04 |  | | acetate kinase |
|  | RS_RS01720 | 1.92 |  | | bacterioferritin-associated ferredoxin protein |
|  | RS_RS11255 | -3.06 |  | | 4-hydroxybenzoate 3-monooxygenase |
|  | RS_RS21405 | 1.55 | 1.93 | | 4-oxalocrotonate decarboxylase |
|  | RS_RS21385 | 1.95 |  | | 2,3-dihydroxy-p-cumate-3,4-dioxygenase (CmtC) (plasmid) |
| Nucleotide metabolism | RS_RS14025 | 1.53 |  | | ribonucleotide-diphosphate reductase subunit alpha |
|  | RS_RS21760 | -2.37 | -2.36 | | anaerobic ribonucleoside triphosphate reductase |
|  | RS_RS21115 |  | -3.29 | | bifunctional nicotinamide-nucleotide adenylyltransferase/Nudix hydroxylase |
|  | RS_RS01000 | -3.71 |  | | phosphoribosylpyrophosphate synthetase |
|  | RS_RS16480 | 1.80 |  | | ATP-dependent DNA helicase Rep |
|  | RS_RS20940 | 1.88 | 2.85 | | error-prone DNA polymerase |
|  | RS_RS20935 | 2.81 | 3.86 | | DNA polymerase IV protein |
|  | RS_RS04735 | 2.55 |  | | ATP-dependent RNA helicase |
|  | RS_RS21835 |  | -1.93 | | molybdopterin-guanine dinucleotide biosynthesis protein B |
|  | RS_RS18505 | 12.36 |  | | GGDEF domain-containing protein |
|  | RS_RS03750 | 1.93 |  | | endonuclease |
|  | RS_RS19210 | 1.69 |  | | phosphoribosyltransferase |
|  | RS_RS04200 | -3.40 |  | | RNA polymerase subunit sigma |
|  | RS_RS07360 | -2.03 |  | | Nucleoside-diphosphate-sugar epimerase |
|  | RS_RS20480 | 2.58 | 2.13 | | RtcB family protein |
| Biosynthesis of other secondary metabolites | RS_RS26360 | 2.42 |  | | hypothethical protein (plasmid) |
|  | RS_RS15890 | 2.33 |  | | hemagglutinin |
|  | RS_RS02540 | 4.82 |  | | bifunctional diguanylate cyclase/phosphodiesterase |
|  | RS_RS20435 |  | -2.43 | | homogentisate 1,2-dioxygenase |
|  | RS_RS18030 | 2.12 |  | | hemagglutinin |
|  | RS_RS17980 | 2.13 | 2.75 | | SAM-dependent methyltransferase |
|  | RS_RS07525 | 1.78 |  | | FkbM family methyltransferase |
|  | RS_RS20835 | -3.33 | -3.24 | | N-acylhomoserine lactone synthase |
|  | RS_RS03905 | -2.73 |  | | hemerythrin |
|  | RS_RS16910 | 1.91 |  | | Panthothenate synthetase |
|  | *acpP* | 1.53 |  | | acyl carrier protein |
|  | RS_RS20895 | -5.12 | -7.18 | | acyl carrier protein |
|  | RS_RS21440 | 1.53 | -2.68 | | hydratase |
|  | RS_RS20190 |  | -1.66 | | non-ribosomal peptide synthase/polyketide synthase |
|  | RS_RS12725 | -3.31 |  | | bile acid:sodium symporter |
|  | RS_RS17725 | -1.72 | -2.43 | | 3-methylitaconate isomerase (plasmid) |
| Metabolism of terpenoids and polyketides | RS_RS12980 | -1.50 |  | | limonene-1,2-epoxide hydrolase |
|  | RS_RS17310 | 2.70 |  | | porin |
|  | RS_RS20570 | -2.45 | | -5.32 | porin |
|  | RS_RS24940 | -2.17 | |  | porin |
|  | RS_RS18245 | -1.92 | |  | feruloyl-CoA synthase |
| Folding, sorting and degradation | RS_RS03230 | -1.86 | |  | molecular chaperone GroEL |
|  | RS_RS00995 | -5.57 | |  | MBL fold metallo-hydrolase |
|  | RS_RS11100 | -5.50 | |  | MBL fold metallo-hydrolase |
|  | RS_RS19960 | 1.64 | | 3.82 | MBL fold metallo-hydrolase |
|  | RS_RS02965 | -1.87 | | -2.08 | phosphohydrolase |
| Hypothetical protein and others | RS_RS21730 | 2.24 | | -4.28 | hypothetical protein |
|  | RS_RS25945 | -2.52 | |  | hypothetical protein |
|  | RS_RS20790 |  | | -3.52 | hypothetical protein |
|  | RS_RS26095 | 1.70 | |  | hypothetical protein |
|  | RS_RS04775 | 1.72 | |  | hypothetical protein |
|  | RS_RS27035 |  | | 1.56 | hypothetical protein |
|  | RS_RS25225 | 2.10 | |  | hypothetical protein |
|  | RS_RS25400 | 2.87 | |  | hypothetical protein |
|  | RS_RS24550 | 3.35 | |  | hypothetical protein |
|  | RS_RS23065 |  | | 3.58 | hypothetical protein |
|  | RS_RS26475 | 3.42 | |  | hypothetical protein |
|  | RS_RS02975 | 3.71 | |  | hypothetical protein |
|  | RS_RS22740 | 3.80 | |  | conserved hypothethical protein (plasmid) |
|  | RS_RS19565 | 3.51 | |  | conserved hypothethical protein (plasmid) |
|  | RS_RS16215 | 3.55 | |  | conserved hypothethical protein |
|  | RS_RS16220 | 12.03 | |  | Conserved hypothethical protein, DNA-binding domain |
|  | RS_RS20885 | -5.14 | | -6.59 | conserved hypothethical protein (plasmid) |
|  | RS_RS20865 | -4.94 | | -6.68 | conserved hypothethical protein (plasmid) |
|  | RS_RS20905 | -2.72 | | -7.21 | conserved hypothethical protein (plasmid) |
|  | RS_RS19245 | -2.31 | | -3.79 | conserved hypothethical protein (plasmid) |
|  | RS_RS20630 | -1.71 | | -4.15 | conserved hypothethical protein (plasmid) |
|  | RS_RS20635 | -1.65 | | -3.35 | conserved hypothethical protein (plasmid) |
|  | RS_RS25495 | -1.58 | |  | conserved hypothethical protein (plasmid) |
|  | RS_RS17965 | 1.59 | | -2.17 | conserved hypothethical protein (plasmid) |
|  | RS_RS20195 | 1.60 | | -5.16 | conserved hypothethical protein (plasmid) |
|  | RS_RS21725 | 1.66 | | -3.25 | Conserved hypothethical protein |
|  | RS_RS20035 | 2.01 | | -4.25 | conserved hypothethical protein, UCP019302 (plasmid) |
|  | RS_RS19220 | 2.16 | |  | conserved hypothethical protein (plasmid) |
|  | RS_RS25135 | 2.33 | |  | conserved hypothethical protein (plasmid) |
|  | RS_RS17970 | 2.56 | | 2.79 | Conserved hypothethical protein |
|  | RS_RS02985 | 2.64 | |  | Conserved hypothethical protein |
|  | RS_RS19545 | 2.79 | |  | conserved hypothethical protein |
|  | RS_RS19550 | 2.80 | |  | conserved hypothethical protein (fragment) |
|  | RS_RS18350 | 2.83 | |  | conserved hypothethical protein (plasmid) |
|  | RS_RS16920 | 2.87 | |  | conserved hypothethical protein |
|  | RS_RS01105 | 3.14 | | 1.96 | conserved hypothethical protein |
|  | RS_RS19115 | 3.23 | |  | conserved hypothethical protein |
|  | RS_RS18945 | 3.40 | |  | conserved hypothethical protein |
|  | RS_RS19570 | 3.61 | -2.82 | | Conserved hypothethical protein |
|  | RS_RS17325 | 2.75 |  | | GALA protein |
|  | RS_RS17400 | 2.15 |  | | Uncharacterised protein |
|  | RS_RS16730 | -3.09 |  | | Uncharacterised protein |
|  | RS_RS25125 | 2.09 |  | | DUF2334 domain-containing protein |
|  | RS_RS09135 |  | 2.15 | | KR domain-containing protein |
|  | RS_RS00900 | -12.99 |  | | DUF2892 domain-containing protein |
|  | RS_RS03840 | -5.82 |  | | DUF3079 domain-containing protein |
|  | RS_RS02970 | -1.89 |  | | conserved protein of unknown function |
|  | RS_RS25420 | 1.59 |  | | conserved protein of unknown function |
|  | RS_RS00955 | -4.07 |  | | conserved protein of unknown function |
|  | RS_RS07520 | 2.02 |  | | TPR domain protein |
|  | RS_RS04415 | 1.95 |  | | HrgA protein |
|  | RS_RS02915 | 1.86 |  | | probable avrd-related protein |
|  | RS_RS08485 | 1.83 |  | | phage portal protein |
|  | RS_RS20330 | 1.81 | 2.03 | | acriflavine resistance protein B |
|  | RS_RS25595 | 1.60 |  | | Hypothethical protein |
|  | RS_RS16195 | 1.71 |  | | Uncharacterized conserved protein |
|  | RS_RS02980 | 1.59 |  | | TonB-dependent receptor |
|  | RS_RS23530 | 1.58 |  | | RidA family protein |
|  | RS_RS23660 | 1.58 |  | | RidA family protein |
|  | RS_RS16205 | 1.53 |  | | phage related protein |
|  | RS_RS04170 | -1.74 |  | | phage related protein |
|  | RS_RS25630 | -1.72 |  | | histone |
|  | RS_RS09685 | 1.50 |  | | phage tail protein |
|  | RS_RS07590 | -3.55 |  | | DUF1488 domain-containing protein |
|  | RS_RS23950 | -3.62 | -3.84 | | FlxA-like protein |
|  | RS_RS07560 | -6.69 |  | | universal stress protein UspA |
|  | RS_RS00885 | -5.89 |  | | universal stress protein UspA |
|  | RS_RS00990 | -5.46 |  | | heat-shock protein Hsp20 |
|  | RS_RS22125 |  | -2.52 | | DUF692 domain-containing protein |
|  | RS_RS24480 |  | -2.03 | | DUF3500 domain-containing protein |
|  | RS_RS20590 | -5.30 | -3.47 | | radical SAM protein |
|  | RS_RS21785 | -4.81 | -4.71 | | SCP2 domain-containing protein |
|  | RS_RS00910 | -3.66 | -1.88 | | universal stress protein UspA |
|  | RS_RS21225 | -3.58 |  | | HrpX protein |
|  | RS_RS00915 | -3.31 |  | | universal stress protein UspA |
|  | RS_RS21330 | -2.97 | -3.52 | | protein PopA1 |
|  | RS_RS21325 | -2.13 |  | | protein PopB |
|  | RS_RS10885 | -2.57 |  | | LamB/YcsF family protein |
|  | RS_RS24350 | -2.00 |  | | SCO family protein |
|  | RS_RS18675 | -1.96 |  | | universal stress protein UspA |
|  | RS_RS24625 | -1.88 |  | | universal stress protein UspA |
|  | RS_RS19310 | -1.86 |  | | Immunity protein 58 |
|  | RS_RS17030 | -1.51 | -3.18 | | flavohemoprotein |
|  | RS_RS03225 | -1.93 |  | | molecular chaperone GroES |
|  | RS_RS18100 | -1.51 | -5.06 | | disulfide bond formation protein B |
|  | RS_RS19240 | -1.76 | -3.23 | | anti-ECFsigma factor, ChrR |
