# Supplementary material for: An anthranilic acid-responsive transcriptional regulator controls the physiology and pathogenicity of Ralstonia solanacearum
Source: PLoS Pathog. 2022 May 26;18(5):e1010562. doi: 10.1371/journal.ppat.1010562 (PMC9176790; doi:10.1371/journal.ppat.1010562)
Supplement: S3 Table — (DOCX) [file ppat.1010562.s017.docx]

**S3** **Table.** Analysis of the homologs of RaaR in various bacteria

| Bacteria | RaaR homologue  Accession No. | RaaR homologue  Identity (%) | TrpE homologue  Identity (%) | TrpG homologue  Identity (%) |
| --- | --- | --- | --- | --- |
| ***Ralstonia*** |  |  |  |  |
| *R. solanacearum* GMI1000 (phylotype Ⅰ) | CAD18063 | 100 | 100 | 100 |
| *R. solanacearum* RS 476 (phylotype Ⅰ) | AST29904 | 100 | 100 | 100 |
| *R. solanacearum* CFBP2957 (phylotype ⅡA) | RCFBP_21162 | 58 | 96 | 95 |
| *R. solanacearum* UW551 (phylotype ⅡB) | RRSL_03337 | 58 | 96 | 95 |
| *R. solanacearum* CMR15 (phylotype Ⅲ) | CMR15_30619 | 58 | 99 | 97 |
| *R. solanacearum* PSI07 (phylotype Ⅵ) | RPSI07_mp0892 | 97 | 97 | 92 |
| *R. insidiosa* ATCC 49129 | ANJ71185 | 57 | 90 | 89 |
| *R. mannitolilytica* SN83A39 | ANA34936 | 58 | 91 | 90 |
| *R. pickettii* 12D | CCA85208 | 57 | 90 | 90 |
| *R. syzygii* | WP_197331713.1 | 59 | 97 | 93 |
| ***Burkholderia*** |  |  |  |  |
| *B. cepacia* | WP_043187611.1 | 74 | 80 | 80 |
| *B. anthina* | WP_175857521.1 | 74 | 79 | 80 |
| *B. insecticola* | WP_016344096.1 | 66 |  |  |
| *B. stagnalis* | WP_124750153.1 | 65 | 79 | 78 |
| *B. multivorans* | WP_035952495.1 | 64 | 79 | 78 |
| *B. latens* | WP_151066912.1 | 64 | 80 | 80 |
| *B. pyrrocinia* | WP_174383536.1 | 64 | 80 | 80 |
| *B. catarinensis* | OJD08338.1 | 63 | 79 | 79 |
| *B. ambifaria* | WP_175759065.1 | 64 | 79 | 79 |
| *B. cenocepacia* | WP_105866151.1 | 64 | 80 | 80 |
| *B. ubonensis* | WP_059758769.1 | 64 | 80 | 79 |
| *B. vietnamiensis* | WP_060122363.1 | 64 | 79 | 80 |
| *B. diffusa* | WP_059913594.1 | 62 | 79 | 79 |
| *B. arboris* | WP_175847395.1 | 63 | 79 | 79 |
| *B. territorii* | WP_059952289.1 | 62 | 79 | 79 |
| *B. oklahomensis* | WP_025989624.1 | 64 | 79 | 80 |
| *B. guangdongensis* | WP_179401125.1 | 64 | 79 | 79 |
| *B. dabaoshanensis* | WP_102645496.1 | 66 | 80 | 82 |
| *B. pseudomallei* | WP_122829568.1 | 63 | 79 | 79 |
| *B. mallei* | WP_120701015.1 | 63 | 79 | 79 |
| *B. gladioli* | WP_186139788.1 | 62 | 80 | 80 |
| *B. plantarii* | KAF1030015.1 | 60 | 80 | 81 |
| ***Paraburkholderia*** |  |  |  |  |
| *P. piptadeniae* | WP_087734211.1 | 66 | 79 | 80 |
| *P. susongensis* | WP_085482355.1 | 66 | 78 | 81 |
| *P. sprentiae* | WP_027199559.1 | 66 | 78 | 80 |
| *P. franconis* | WP_152761501.1 | 66 | 80 | 79 |
| *P. fungorum* | PZR46891.1 | 65 | 80 | 81 |
| *P. diazotrophica* | WP_090863175.1 | 65 | 80 | 80 |
| *P. insulsa* | WP_030099966.1 | 65 | 79 | 80 |
| *P. caffeinilytica* | WP_115781709.1 | 65 | 79 | 80 |
| *P. agricolaris* | WP_153134371.1 | 66 |  | 80 |
| *P. aspalathi* | WP_093645641.1 | 66 | 79 | 81 |
| *P. aromaticivorans* | WP_095417618.1 | 64 | 79 | 81 |
| *P. caballeronis* | WP_134166843.1 | 67 | 80 | 80 |
| *P. xenovorans* | WP_011486626.1 | 66 | 80 | 81 |
| *P. phymatum* | WP_012399636.1 | 64 | 80 | 79 |
| *P. sediminicola* | WP_175051933.1 | 65 | 79 | 81 |
| *P. phytofirmans* | WP_063494615.1 | 65 | 80 | 81 |
| *P. dipogonis* | WP_134457248.1 | 65 | 79 | 80 |
| *P. azotifigens* | WP_147233982.1 | 65 | 80 | 80 |
| *P. ginsengisoli* | WP_042328695.1 | 64 | 79 | 81 |
| *P. panacisoli* | WP_149668557.1 | 65 | 79 | 81 |
| *P. phenoliruptrix* | WP_015001498.1 | 62 | 79 | 80 |
| *P. silvatlantica* | WP_110383036.1 | 67 | 79 | 79 |
| *P. nodosa* | WP_028201792.1 | 66 | 79 | 79 |
| *P. heleia* | WP_042265655.1 | 66 | 79 | 79 |
| *P. acidipaludis* | WP_027793351.1 | 64 | 79 | 80 |
| *P. terrae* | WP_042306384.1 | 64 | 79 | 80 |
| *P. acidisoli* | WP_158947955.1 | 66 | 78 | 81 |
| *P. lycopersici* | WP_091993674.1 | 66 | 79 | 79 |
| *P. sartisoli* | WP_090527529.1 | 63 | 78 | 79 |
| *P. unamae* | WP_112170248.1 | 66 | 79 | 79 |
| *P. dilworthii* | WP_027802526.1 | 64 | 79 | 81 |
| *P. kururiensis* | WP_042299372.1 | 62 | 80 | 83 |
| *P. ferrariae* | WP_028227743.1 | 66 | 79 | 80 |
| *P. phenazinium* | WP_074263220.1 | 65 | 79 | 80 |
| *P. solisilvae* | WP_175111237.1 | 64 | 78 | 79 |
| *P. bannensis* | WP_027815746.1 | 65 | 79 | 80 |
| *P. humisilvae* | WP_175229980.1 | 63 | 78 | 79 |
| *P. rhizosphaerae* | WP_134190690.1 | 62 | 79 | 79 |
| ***Caballeronia*** |  |  |  |  |
| *C. calidae* | WP_062611549.1 | 75 | 78 | 80 |
| *C. ptereochthonis* | SAK84133.1 | 65 | 79 | 79 |
| *C. hypogeia* | WP_061165610.1 | 65 | 79 | 78 |
| *C. turbans* | SAL20198.1 | 65 | 79 | 79 |
| *C. pedi* | WP_061172651.1 | 65 | 79 | 77 |
| *C. peredens* | SAL26377.1 | 65 | 79 | 78 |
| *C. glebae* | WP_086966015.1 | 63 | 78 | 77 |
| *C. jiangsuensis* | KAK49509.1 | 65 | 78 | 77 |
| *C. grimmiae* | WP_035961865.1 | 64 | 79 | 78 |
| *C. terrestris* | WP_087655775.1 | 62 | 78 | 78 |
| *C. sordidicola* | WP_089165581.1 | 62 | 78 | 81 |
| *C. udeis* | SAL61424.1 | 62 | 78 | 79 |
| *C. choica* | WP_087646003.1 | 62 | 78 | 79 |
| *C. mineralivorans* | WP_047895543.1 | 62 | 78 | 79 |
| ***Trinickia*** |  |  |  |  |
| *T. symbiotica* | WP_018443243.1 | 62 | 79 | 79 |
| *T. fusca* | WP_121277651.1 | 63 | 80 | 80 |
| *T. soli* | WP_102609788.1 | 65 | 79 | 80 |
| *T. caryophylli* | WP_085227782.1 | 61 | 81 | 77 |
| *T. diaoshuihuensis* | WP_116137112.1 | 65 | 80 | 79 |
| *T. dinghuensis* | WP_115532624.1 | 60 | 79 | 82 |
| ***Pandoraea*** |  |  |  |  |
| *P. thiooxydans* | WP_047215167.1 | 60 | 81 | 77 |
| *P. norimbergensis* | WP_058377048.1 | 60 | 78 | 80 |
| *P. captiosa* | WP_150624658.1 | 60 | 79 | 80 |
| *P. pnomenusa* | WP_063598832.1 | 58 | 79 | 80 |
| *P. sputorum* | WP_039395306.1 | 60 | 79 | 80 |
| *P. morbifera* | WP_150565864.1 | 58 | 80 | 80 |
| *P. apista* | AJE98606.1 | 58 | 78 | 79 |
| *P. vervacti* | WP_044453669.1 | 59 | 79 | 80 |
| *P. faecigallinarum* | WP_047906757.1 | 60 | 79 | 79 |
| *P. pulmonicola* | AJC20835.1 | 58 | 80 | 81 |
| *P. nosoerga* | WP_150553923.1 | 58 | 80 | 78 |
| *P. commovens* | WP_150664102.1 | 59 | 79 | 78 |
| *P. eparura* | WP_150588480.1 | 57 | 79 | 80 |
| *P. aquatica* | WP_150575327.1 | 59 | 79 | 78 |
| *P. oxalativorans* | WP_046290605.1 | 58 | 79 | 78 |
| *P. fibrosis* | VVD93477.1 | 59 | 80 | 78 |
| *P. terrigena* | WP_150611236.1 | 58 | 79 | 80 |
| *P. anhela* | WP_150667462.1 | 59 | 79 | 80 |
| *P. capi* | WP_150720984.1 | 59 | 79 | 80 |
| *P. anapnoica* | WP_150736591.1 | 58 | 80 | 78 |
| *P. cepalis* | WP_150562235.1 | 57 | 79 | 80 |
| *P. communis* | WP_150583398.1 | 57 | 80 | 80 |
| *P. horticolens* | WP_150618734.1 | 57 | 80 | 80 |
| *P. soli* | WP_150550903.1 | 56 | 79 | 80 |
| ***Cupriavidus*** |  |  |  |  |
| *C. gilardii* | WP_174108260.1 | 57 | 86 | 82 |
| *C. pinatubonensis* JMP134 | AAZ59465.1 | 57 | 86 | 83 |
| *C. necator* | WP_153946178.1 | 57 | 86 | 84 |
| *C. oxalaticus* | WP_063239917.1 | 56 | 86 | 80 |
| *C. nantongensis* | WP_062796190.1 | 57 | 86 | 83 |
| *C. alkaliphilus* | WP_092315226.1 | 56 | 85 | 83 |
| *C. lacunae* | WP_115212032.1 | 56 | 86 | 82 |
| *C. taiwanensis* | WP_116381679.1 | 56 | 85 | 83 |
| *C. pinatubonensis* | WP_140950888.1 | 56 | 86 | 83 |
| *C. metallidurans* CH34 | ABF06944.1 | 56 | 86 | 84 |
| *C. basilensis* OR16 | EHP39988.1 | 55 | 86 | 83 |
| *C. pauculus* | WP_150373196.1 | 55 | 85 | 83 |
| ***Aromatoleum*** |  |  |  |  |
| *A. tolulyticum* | WP_076602590.1 | 45 | 59 |  |
| *A. evansii* | WP_169129739.1 | 44 | 58 |  |
| *A. toluclasticum* | WP_018990839.1 | 44 | 58 |  |
| *A. diolicum* | WP_169262114.1 | 44 | 59 |  |
| *A. toluvorans* | WP_169257362.1 | 45 | 58 |  |
| *A. petrolei* | WP_169205273.1 | 44 | 58 |  |
| ***Herbaspirillum*** |  |  |  |  |
| *H. autotrophicum* | WP_050464310.1 | 47 | 76 | 74 |
| *H. lusitanum* | WP_016832549.1 | 49 | 77 | 76 |
| *H. hiltneri* | WP_144969265.1 | 49 | 77 | 75 |
| *H. rhizosphaerae* | WP_050479106.1 | 50 | 76 |  |
| *H. rubrisubalbicans* | WP_058897418.1 | 46 | 77 | 75 |
| *H. seropedicae* | WP_013236684.1 | 49 | 76 | 75 |
| *H. robiniae* | WP_079217443.1 | 47 | 76 | 75 |
| *H. frisingense* | KAF1046794.1 | 47 | 77 | 75 |
| *H. chlorophenolicum* | WP_050468620.1 | 46 | 76 | 74 |
| *H. huttiense* | MBN9358194.1 | 45.7 | 76 | 75 |
| ***Massilia*** |  |  |  |  |
| *M. violacea* | WP_183439311.1 | 51 | 76 | 78 |
| *M. namucuonensis* | WP_093553799.1 | 49 | 76 | 77 |
| *M. guangdongensis* | WP_161024126.1 | 48 | 76 | 76 |
| *M. glaciei* | WP_106760380.1 | 48 | 75 |  |
| *M. eurypsychrophila* | WP_099790926.1 | 46 | 74 |  |
| ***Duganella*** |  |  |  |  |
| *D. levis* | WP_161055675.1 | 49 | 75 | 76 |
| *D. ginsengisoli* | WP_170305630.1 | 49 | 76 | 76 |
| *D. radicis* | WP_155464040.1 | 51 | 75 | 75 |
| *D. sacchari* | WP_072783122.1 | 49 | 75 | 77 |
| *D. pernnla* | WP_161043138.1 | 49 | 75 | 76 |
| ***Noviherbaspirillum*** |  |  |  |  |
| *N. malthae* | WP_194720476.1 | 53 | 78 | 76 |
| *N. aerium* | WP_151639396.1 | 52 | 79 | 77 |
| *N. galbum* | WP_163962530.1 | 51 | 79 | 76 |
| *N. massiliense* | WP_019142158.1 | 49 | 78 | 77 |
| *N. autotrophicum* | WP_040041634.1 | 51 | 78 | 76 |
| *N. denitrificans* | WP_088706336.1 | 49 | 78 | 76 |
| ***Sneathiella*** |  |  |  |  |
| *S. limimaris* | WP_169570128.1 | 48 |  |  |
| *S. aquimaris* | WP_169543034.1 | 44 |  |  |
| *S. chinensis* | WP_169560313.1 | 44 |  |  |
| ***Azospirillum*** |  |  |  |  |
| *A. doebereinerae* | WP_127001121.1 | 46 |  |  |
| *A. lipoferum* | WP_085556589.1 | 45 |  |  |
| *A. thiophilum* | WP_045580046.1 | 44 |  |  |
| *A. lipoferum* | WP_149230305.1 | 44 |  |  |
| *A. melinis* | WP_174469852.1 | 44 |  |  |
| *A. oryzae* | WP_149196912.1 | 44 |  |  |
| ***Achromobacter*** |  |  |  |  |
| *A. insolitus* | WP_175201134.1 | 73 | 69 |  |
| *A. xylosoxidans* ATCC 27061 | AHC49211.1 | 72 | 69 |  |
| *A. aegrifaciens* | WP_054457920.1 | 49 | 69 |  |
| ***Collimonas*** |  |  |  |  |
| *C. arenae* | WP_144738231.1 | 50 | 77 | 76 |
| *C. fungivorans* | WP_120234087.1 | 50 | 79 | 77 |
| *C. pratensis* | WP_168231098.1 | 49 | 78 | 76 |
| ***Mycetohabitans*** |  |  |  |  |
| *M. endofungorum* | WP_104076532.1 | 75 | 77 | 75 |
| *M. rhizoxinica* | WP_013436429.1 | 74 | 78 | 75 |
| ***Glaciimonas*** |  |  |  |  |
| *G. immobilis* | WP_168056329.1 | 50 | 76 | 78 |
| *G. soli* | WP_153234156.1 | 47 | 76 | 78 |
| ***Pseudoduganella*** |  |  |  |  |
| *P. violaceinigra* | WP_035374625.1 | 49 | 76 | 76 |
| *P. eburnea* | WP_155454929.1 | 48 | 76 | 77 |
| ***Usitatibacter*** |  |  |  |  |
| *U. palustris* | WP_171160898.1 | 46 | 57 |  |
| *U. rugosus* | WP_171090052.1 | 46 | 57 |  |
| ***Azoarcus*** |  |  |  |  |
| *A. olearius* | WP_011764065.1 | 45 | 58 |  |
| *A. halotolerans* | WP_159692588.1 | 44 | 59 |  |
| ***Silvimonas*** |  |  |  |  |
| *S. iriomotensis* | GGP22785.1 | 43 | 63 | 71 |
| *S. terrae* | WP_184100700.1 | 43 | 63 |  |
| ***Andreprevotia*** |  |  |  |  |
| *A. lacus* | WP_084088956.1 | 44 | 63 | 74 |
| *A. chitinilytica* | WP_169741324.1 | 44 | 63 | 75 |
| ***Others*** |  |  |  |  |
| *Bacillus pumilus* | WP_137055785.1 | 65 |  |  |
| *Pantoea rwandensis* | WP_084934091.1 | 61 |  |  |
| *Burkholderiaceae bacterium* 26 | KJK04521.1 | 58 | 95 | 93 |
| *Robbsia andropogonis* | WP_024905136.1 | 51 | 75 | 76 |
| *Burkholderiales bacterium* | MBI3285646.1 | 50 | 72 | 69 |
| *Limnohabitans planktonicus* | WP_053169317.1 | 50 | 70 | 68 |
| *Oxalobacteraceae bacterium* OM1 | TFW03511.1 | 50 | 74 | 77 |
| *Sterolibacteriaceae bacterium* | MBK8336555.1 | 50 | 59 |  |
| *Janthinobacterium lividum* | WP_128140660.1 | 49 | 75 | 77 |
| *Rugamonas rubra* | WP_093389203.1 | 49 | 75 | 77 |
| *Bordetella genomo* sp. 11 | WP_094843734.1 | 48 | 70 |  |
| *Rhodospirillales bacterium* | MBL8705517.1 | 46 |  |  |
| *Betaproteobacteria bacterium* GR16-43 | APV52161.1 | 46 | 78 | 67 |
| *Sphingomonadales bacterium* | NJO24034.1 | 46 |  |  |
| *Undibacterium parvum* | WP_126126986.1 | 49 |  |  |
| *Rhizobiales bacterium* | HER01996.1 | 45 | 71 |  |
| *Jeongeupia chitinilytica* | WP_189462325.1 | 45 | 62 | 76 |
| *Rhodospirillaceae bacterium* | MBV38215.1 | 45 |  |  |
| *Proteobacteria bacterium* | TDJ66958.1 | 44 | 60 | 69 |
| *Alphaproteobacteria bacterium* HT1-32 | MRG70659.1 | 44 | 68 |  |
| *Chitinimonas taiwanensis* | MBB5025236.1 | 43 | 64 |  |
| *Candidatus Accumulibacter* sp. SK-12 | EXI69728.1 | 43 | 58 | 70 |
| *Niveibacterium umoris* | WP_183638422.1 | 42 | 57 |  |
| *Propionivibrio dicarboxylicus* | WP_091938390.1 | 42 | 59 |  |
| *Terasakiella brassicae* | WP_188665577.1 | 42 |  |  |
