# Supplementary material for: An anthranilic acid-responsive transcriptional regulator controls the physiology and pathogenicity of Ralstonia solanacearum
Source: PLoS Pathog. 2022 May 26;18(5):e1010562. doi: 10.1371/journal.ppat.1010562 (PMC9176790; doi:10.1371/journal.ppat.1010562)
Supplement: S4 Table — (DOCX) [file ppat.1010562.s018.docx]

**S4 Table.** Bacterial strains and plasmids used in this study

| **Strain or plasmid** | **Phenotype and/or characteristic(s)** | **Source or reference** |
| --- | --- | --- |
| ***R. solanacearum*** |  |  |
| GMI1000 | Wild-type strain of *R. solanacearum* | ATCCBAA-1114 |
| Δ*trpEG* | Anthranilic acid-minus mutant derived from GMI1000 with *trpEG* being deleted | [1] |
| Δ*trpEG*(*raaR*) | Mutant Δ*trpEG* harboring the expression construct pBBRI-2-*raaR* | This study |
| Δ*raaR* | Deletion mutant derived from GMI1000 with *raaR* being deleted | This study |
| Δ*trpEG*Δ*raaR* | Deletion mutant derived from GMI1000 with *trpEG* and *raaR* being deleted | This study |
| Δ*raaR*(*raaR*) | Mutant Δ*raaR* harboring the expression construct pBBRI-2-*raaR* | This study |
| Δ*trpEG*(*RSp0942*) | Mutant Δ*trpEG* harboring the expression construct pBBRI-2-*RSp0942* | This study |
| Δ*trpEG*(*RSc0542*) | Mutant Δ*trpEG* harboring the expression construct pBBRI-2-*RSc0542* | This study |
| Δ*trpEG*(*RSc0615*) | Mutant Δ*trpEG* harboring the expression construct pBBRI-2-*RSc0615* | This study |
| Δ*trpEG*(*RSc1110*) | Mutant Δ*trpEG* harboring the expression construct pBBRI-2-*RSc1110* | This study |
| Δ*trpEG*(*RSc1880*) | Mutant Δ*trpEG* harboring the expression construct pBBRI-2-*RSc1880* | This study |
| Δ*trpEG*(*RSc1472*) | Mutant Δ*trpEG* harboring the expression construct pBBRI-2-*RSc1472* | This study |
| Δ*trpEG*(*RSc2537*) | Mutant Δ*trpEG* harboring the expression construct pBBRI-2-*RSc2537* | This study |
| Δ*trpEG*(*RSc2761*) | Mutant Δ*trpEG* harboring the expression construct pBBRI-2-*RSc2761* | This study |
| Δ*trpEG*(*RSc3332*) | Mutant Δ*trpEG* harboring the expression construct pBBRI-2-*RSc3332* | This study |
| Δ*raaR*(*mvfR*) | Mutant Δ*raaR* harboring the expression construct pBBRI-2-*mvfR* | This study |
| GMI1000(P*phcB-lacZ*) | GMI1000 harboring the reporter construct P*phcB-lacZ* | This study |
| Δ*raaR*(P*phcB-lacZ*) | Δ*raaR* harboring the reporter construct P*phcB-lacZ* | This study |
| Δ*trpEG*Δ*raaR*(P*phcB-lacZ*) | Δ*trpEG*Δ*raaR* harboring the reporter construct P*phcB-lacZ* | This study |
| GMI1000(P*solI-lacZ*) | GMI1000 harboring the reporter construct P*solI-lacZ* | This study |
| Δ*raaR*(P*solI-lacZ*) | Δ*raaR* harboring the reporter construct P*solI-lacZ* | This study |
| Δ*trpEG*Δ*raaR*(P*solI-lacZ*) | Δ*trpEG*Δ*raaR* harboring the reporter construct P*solI-lacZ* | This study |
| GMI1000(P*raaR-lacZ*) | GMI1000 harboring the reporter construct P*raaR-lacZ* | This study |
| Δ*trpEG*(P*raaR-lacZ*) | Δ*trpEG* harboring the reporter construct P*raaR-lacZ* | This study |
| GMI1000(P*gyrB-lacZ*) | GMI1000 harboring the reporter construct P*gyrB-lacZ* | This study |
| Δ*raaR*(P*gyrB-lacZ*) | Δ*raaR* harboring the reporter construct P*gyrB-lacZ* | This study |
| GMI1000(P*epsA-lacZ*) | GMI1000 harboring the reporter construct P*epsA-lacZ* | [1] |
| Δ*raaR*(P*epsA-lacZ*) | GMI1000 harboring the reporter construct P*epsA-lacZ* | This study |
| ***Escherichia coli*** |  |  |
| DH5α | *supE44 lacU169(80lacZM15) hsdR17 recA1 endA1 gyrA96 thi-1 relA1 pir* | Laboratory collection |
| BL21 | *F-ompT hsdS (rB-mB-) dcm+ Tetr gal (DE3) endA* | Laboratory collection |
| **Plasmid** |  |  |
| pK18 | pK18, sacB^+^; gene replacement vector | Laboratory collection |
| pK18-*raaR* | pK18 containing fragments flanking *raaR* | This study |
| pK18-*trpEG* | pK18 containing fragments flanking *trpEG* | [1] |
| pBBRI-2 | Broad host range cloning vector | Laboratory collection |
| pBBRI-2-*raaR* | pBBRI-2 containing *raaR* | This study |
| pBBRI-2-*RSp0942* | pBBRI-2 containing *RSp0942* | This study |
| pBBRI-2-*RSc0542* | pBBRI-2 containing *RSc0542* | This study |
| pBBRI-2-*RSc0615* | pBBRI-2 containing *RSc0615* | This study |
| pBBRI-2-*RSc1110* | pBBRI-2 containing *RSc1110* | This study |
| pBBRI-2-*RSc1880* | pBBRI-2 containing *RSc1880* | This study |
| pBBRI-2-*RSc1472* | pBBRI-2 containing *RSc1472* | This study |
| pBBRI-2-*RSc2537* | pBBRI-2 containing *RSc2537* | This study |
| pBBRI-2-*RSc2761* | pBBRI-2 containing *RSc2761* | This study |
| pBBRI-2-*RSc3332* | pBBRI-2 containing *RSc3332* | This study |
| pBBRI-2-*mvfR* | pBBRI-2 containing *mvfR* | This study |
| pMAL-c5X | Expression vector | Laboratory collection |
| pMAL-c5X-*raaR* | pMAL-c5X containing *raaR* | This study |
| pMAL-c5X-LysR_substrate(*raaR*) | pMAL-c5X containing LysR substrate binding domain of RaaR | This study |
| pMAL-c5X-*mvfR* | pMAL-c5X containing *mvfR* | This study |
| pMAL-c5X-*raaR^A171F^* | pMAL-c5X containing *raaR^A171F^* | This study |
| pMAL-c5X-*raaR^L191A^* | pMAL-c5X containing *raaR^L191A^* | This study |
| pMAL-c5X-*raaR^L192A^* | pMAL-c5X containing *raaR^L192A^* | This study |
| pMAL-c5X-*raaR^I249A^* | pMAL-c5X containing *raaR^I249A^* | This study |
| pME2-*lacZ* | Broad-host-range cloning vector | Laboratory collection |
| P*phcB*-*lacZ* | pME2-*lacZ* containing the promoter of *phcB* | This study |
| P*solI*-*lacZ* | pME2-*lacZ* containing the promoter of *solI* | This study |
| P*epsA*-*lacZ* | pME2-*lacZ* containing the promoter of *epsA* | [1] |
| P*gyrB*-*lacZ* | pME2-*lacZ* containing the promoter of *gyrB* | This study |
| P*raaR*-*lacZ* | pME2-*lacZ* containing the promoter of *raaR* | This study |

**References**

1. Song S, Yin W, Sun X, Cui B, Huang L, Li P, et al. Anthranilic acid from *Ralstonia solanacearum* plays dual roles in intraspecies signalling and inter-kingdom communication. ISME J. 2020; 14(9):2248-2260. https://doi.org/10.1038/s41396-020-0682-7
