# Supplementary material for: An anthranilic acid-responsive transcriptional regulator controls the physiology and pathogenicity of Ralstonia solanacearum
Source: PLoS Pathog. 2022 May 26;18(5):e1010562. doi: 10.1371/journal.ppat.1010562 (PMC9176790; doi:10.1371/journal.ppat.1010562)
Supplement: S5 Table — (DOCX) [file ppat.1010562.s019.docx]

**S5** **Table.** PCR primers used in this study

| **Primer** | **Sequence (5’-3’)** |
| --- | --- |
| For deletion |  |
| *raaR*L-F | CCGGAATTCAATGTAAAGCGCGTCGATAT |
| *raaR*L-R | GTTCATCAGGGCTTCGAACATCTTGAGGG |
| *raaR*R-F | GTTCGAAGCCCTGTATGAACGCAGCCCCAC |
| *raaR*R-R | CCCAAGCTTCACTCCATTCCCGAAGGCAT |
| *trpEG*L-F | CGGGATCCCCTGATGTTCTGCTAACGT |
| *trpEG*L-R | TAGGCAAATGCCGATGTACTCCTTGGCCTTGAG |
| *trpEG*R-F | GAGTACATCGGCATTTGCCTACCGGTGCTG |
| *trpEG*R-R | CCAAGCTTATGATCTCGCCGTCTTCGGT |
| For *in trans* expression |  |
| *raaR*-F | CCCAAGCTTATGGACTTGCGAAAACTGCC |
| *raaR*-R | CCGGAATTCCTATGCCGCGCGGCCGG |
| *RSp0942*-F | CCCAAGCTTTTGAACTTCAGGCGATTGC |
| *RSp0942*-R | CGCGGATCCTTATGACACCTCGTGGTGCT |
| *RSc0542*-F | CCCAAGCTTATGGATCTCAACCAACTGGC |
| *RSc0542*-R | CGCGGATCCTCATGCCGGATCGCTG |
| *RSc0615*-F | CCGGAATTCATGACACCGCAAGATCTG |
| *RSc0615*-R | CGCGGATCCTCAGATCGGCCCCGAC |
| *RSc1110*-F | CCCAAGCTTATGGATAACAAGGTGCCACG |
| *RSc1110*-R | CGCGGATCCCTAGCCCTTCGTGCGATTTT |
| *RSc1880*-F | CCCAAGCTTATGGAACTCCGACAACTCGAA |
| *RSc1880*-R | CCGGAATTCTCATGCGGACGCGCCTT |
| *RSc1472*-F | CCCAAGCTTATGCGAACGACGGACTGGAA |
| *RSc1472*-R | CGCGGATCCTCAAAGCAGGGCCGCG |
| *RSc2537*-F | CCCAAGCTTATGAATGTGACGCTGCGC |
| *RSc2537*-R | CGCGGATCCCTAGCCGGCCGGCAG |
| *RSc2761*-F | CCCAAGCTTATGCCGAGACGCCTCCCT |
| *RSc2761*-R | CGCGGATCCTCAAAACACCTCGACCGGG |
| *RSc3332*-F | CCCAAGCTTATGGACAAGCTGCGCAGC |
| *RSc3332*-R | CGCGGATCCCTAGGTCAATCCTCTTGCTG |
| *mvfR*-F | CCCAAGCTTATGCCTATTCATAACCTGAATC |
| *mvfR*-R | CCGGAATTCCTACTCTGGTGCGGCGCG |
| For protein expression |  |
| RaaR-MBP-F | CCGGAATTCATGGACTTGCGAAAACTGCC |
| RaaR-MBP-R | CCCAAGCTTCTATGCCGCGCGGCCGG |
| RaaR^A171F^-F | GTTGAACGTCGG**AAA**GCAGGCGGGGAA |
| RaaR^A171F^-R | TTCCCCGCCTGC**TTT**CCGACGTTCAAC |
| RaaR^L191A^-F | GTCGCTGCGCAG**GCG**CGTCACGTCGGC |
| RaaR^L191A^-R | GCCGACGTGACG**CGC**CTGCGCAGCGAC |
| RaaR^L192A^-F | GTAGTCGCTGCG**GCG**CAACGTCACGTC |
| RaaR^L192A^-R | GACGTGACGTTG**CGC**CGCAGCGACTAC |
| RaaR^I249A^-F | GAGCCGTCCGTT**GCG**GATCTCCTGCAT |
| RaaR^I249A^-R | ATGCAGGAGATC**CGC**AACGGACGGCTC |
| LysR_substrate(*raaR*)-MBP-F | CCGGAATTCCGCTACGCCGAGCGGATC |
| LysR_substrate(*raaR*)-MBP-R | CCCAAGCTTCTATGCCGCGCGGCCGG |
| MvfR-MBP-F | CCGGAATTCATGCCTATTCATAACCTGAATC |
| MvfR-MBP-R | CCCAAGCTTCTACTCTGGTGCGGCGCG |
| For EMSA assay |  |
| EMSA-*phcB*F | GACGCCCTTGACCACGCT |
| EMSA-*phcB*R | TGCCGGATCGATTTGGTTG |
| EMSA-mutant *phcB*R | CACCCGGAACGGTCCCGGTCTGGCGGCGTG |
| EMSA-mutant *phcB*F | GACCGGGACCGTTCCGGGTGCATGGTAGTG |
| EMSA-*solI*F | TGATGCTTCGATGGCGAC |
| EMSA-*solI*R | GCCGCCATGAATGAATGTCT |
| EMSA-mutant *solI*R | GGGTGCGATGGGCGGCGATCGCGTAGCCTC |
| EMSA-mutant *solI*F | CGATCGCCGCCCATCGCACCCTGTCAATCCT |
| EMSA-*epsA*F | ACTTTTAAGAAATCGAATTC |
| EMSA-*epsA*R | TGCCATATTCGGAATGCTTA |
| EMSA-mutant *epsA*R | GTGCGTTATTAGCGTCAATTCGATACAGAA |
| EMSA-mutant *epsA*F | AATTGACGCTAATAACGCACCCAACTGTAA |
| EMSA-*gyrB*F | CCTCGAACCAGGTCAAGTTC |
| EMSA-*gyrB*R | TGTGGATTGCGGTTTCTGCT |
| For reporter |  |
| P*phcB*-F | CCCAAGCTTGACGCCCTTGACCACGCT |
| P*phcB*-R | CCGGAATTCTGCCGGATCGATTTGGTTG |
| P*solI*-F | CCCAAGCTTTGATGCTTCGATGGCGAC |
| P*solI*-R | CCGGAATTCGCCGCCATGAATGAATGTCT |
| p*epsA*-F | CCGCTCGAGCCTGCCAGCCTGTAACGC |
| p*epsA*-R | CCGCTCGAGCACGCTCAACGACACGACT |
| P*gyrB*-F | CCCAAGCTTCCTCGAACCAGGTCAAGTTC |
| P*gyrB*-R | CCGGAATTCTGTGGATTGCGGTTTCTGCT |
| P*raaR*-F | CCCAAGCTTAGCCGTTCGACGTCCACC |
| P*raaR*-R | CCGCTCGAGGCCGAGATTGGGCAGTTTTC |
