# Supplementary material for: An anthranilic acid-responsive transcriptional regulator controls the physiology and pathogenicity of Ralstonia solanacearum
Source: PLoS Pathog. 2022 May 26;18(5):e1010562. doi: 10.1371/journal.ppat.1010562 (PMC9176790; doi:10.1371/journal.ppat.1010562)
Supplement: Attachment — Submitted filename: Response to Reviewers.docx [file ppat.1010562.s020.docx]

Dear Editor,

**RE: PPATHOGENS-D-21-02479**

We thank you and the reviewers for the valuable comments and suggestions, which have been followed in the revision of our manuscript “**An anthranilic acid-responsive transcriptional regulator controls the physiology and pathogenicity of *Ralstonia solanacearum***”. As suggested, we have added more experiments to confirm the relationship between RaaR and anthranilic acid (**Fig. 2, Fig. 3A, Fig. S3, Fig. S6, Fig. S7, Fig. S12**). We have also moved the method section in the supplementary information to the main text. In our previous study (Song *et al*., *ISMEJ*, 2020), we have conducted the experiment to restore ΔtrpEG with exogenous anthranilic acid in **Fig. 6**. In addition, we have added the pictures of infected and control plants in **Fig. 6A**, and moved **Fig. S1** to the main text as suggested. You will find the detailed point-to-point response below. We hope that you will find our revised manuscript in this version suitable for publication in the *PLOS Pathogens*.

Sincerely yours,

Yinyue Deng

**Point-to-point response to reviewers’ suggestions**

**Part I - Summary**

Concerns

One area where this paper falls short is in examining the breadth of the *R. solanacearum* species complex. When they search for RaaR homologs in Table S2, it would be useful to show if RaaR is conserved across Rs phylotypes. They could include comparisons to strains like CFBP2957 (Phylotype II), UW551 (Phylotype II), CMR15 (Phylotype III), PSI07 (Phylotype VI), or other strains with available genome sequences. The authors also suggest in the discussion that because they can detect anthranilic acid in some Rs hosts but not in non-host maize or rice, anthranilic acid may help Rs with host sensing. However, the three hosts they test are all *solanaceous* crops (tomato, eggplant, and pepper) and do not capture the diversity of the Rs host range. Some individual strains, including GMI1000, can have extremely wide host ranges. It would be better to include a wider variety of Rs hosts in this test, including monocot hosts like banana and ginger. However, this experiment is not vital to the main focus of the manuscript and could be simply removed. If this supplemental figure is included, the authors should also provide more detail in how they sampled tissues from the tested crops.

Response: Thank you very much for the good suggestions. We have searched and added the RaaR homologs of other Rs phylotypes, including in CFBP2957 (Phylotype II), UW551 (Phylotype II), CMR15 (Phylotype III), PSI07 (Phylotype VI) in **Table S3**. We have also tested anthranilic acid in some more hosts as suggested, including banana, ginger and mulberry, and found that these hosts also have this compound as shown in the Figure below. As this experiment is not vital to the manuscript, so we removed this figure as suggested.

Figure. Detection of anthranilic acid concentration in the roots and stems of the host (Tomato, Eggplant, Pepper, Ginger, Mulberry and Banana) and non-host (Maize, Rice and Wheat) plants of *R. solanacearum*.

Reviewer #3: In this manuscript “A responsive transcriptional regulator controls *Ralstonia solanacearum* pathogenicity by sensing anthranilic acid” (PPATHOGENS-D-21-02479) the authors identified a new signaling system formed by one-component system RaaR (LysR-type regulator) and signal molecule anthranilic acid. RaaR-anthranilic acid complex was verified by ITC and CD experiments. Also, it was suggested that anthranilic acid binding caused conformational changes in RaaR, which positively affected its interaction with target promoters. I have just a few suggestions to reinforce these interesting findings and clarify some parts in text.

Response: Thank you very much for the good suggestions.

**Part II – Major Issues: Key Experiments Required for Acceptance**

Reviewer #1: 1. The major weakness of the paper is that it does not provide sufficient proof that RaaR act downstream to anthranilic acid and not in parallel to it. If RaaR regulate QS signal synthesis, its overexpression might rescue the *trpGE* phenotypes regardless if it act downstream to it or not. To clearly demonstrate that RaaR and RrpGE function in the same pathway, epistasis experiments in the presence and absence of anthranilic acid are required:

a. The authors should produce a *raaR*/*trpGE* double mutant and examine if their phenotypes (*phcB* and *solI* expression by LacZ promter fusions, motility, exoenzyme activity) are additive or not.

b. The authors should try to rescue these phenotypes by exogenous addition of anthranilic acid (as conducted in fig 6 in authors ISME paper): If indeed RaaR act downstream to TrpGE, exogenous addition of anthranilic acid will rescue the *trpGE* mutant but not the *trpGE*/*raaR* double mutant.

Response: Many thanks for your good suggestions. We have constructed the *trpEG* and *raaR* double deletion mutant, and we found that double deletion of *trpEG* and *raaR* caused the same phenotypic changes as observed with the ΔtrpEG mutant (**Fig. 2**). And the addition of exogenous anthranilic acid couldn’t restore these phenotypes of the *raaR* deletion mutant and the *trpEG* and *raaR* double deletion mutant to the wild-type strain levels (**Fig. 2**). We have also tested the expression levels of both *phcB* and *solI* in the *trpEG* and *raaR* double deletion mutant. The results show that double deletion of *trpEG* and *raaR* and single deletion of *raaR* reduce the expression levels of both *phcB* and *solI* at the similar levels (**Fig. 3A**)

2. Some of the experiments described in the manuscript are lacking proper controls:

a. The authors demonstrated that RaaR binds to the *phcB*, *solI* and *espA* promoters in vitro by EMSA. Many transcriptional regulators harbors general affinity to DNA in *in vitro*, which results in high false positive rate in EMSA analysis when conducted without proper controls. As far as I see, all EMSA experiments used with RaaR in this study provided positive results, which make one wonder how specific is the binding of RaaR to these particular promoters. To address this issue the authors should repeat the EMSA experiments using promoters of genes that are NOT directly regulated by RaaR and show that RaaR demonstrate high affinity to *phcB*, *solI* and *espA* promoters and not others.

b. To make sure that RaaR does not harbor a pleiotropic effect on protein expression or LacZ activity, the authors should use a negative control (maybe a promotor of a housekeeping gene fused to LacZ) in their promoter LacZ fusion assays.

c. Figure 5. The authors should conduct their binding assays with one of the structural analogs of anthranilic acid that were used in fig S8 as a negative control.

Response: Thanks a lot for your good suggestion. We have selected a housekeeping gene *gyrB*, which encodes DNA topoisomerase (ATP-hydrolyzing) subunit B, and used it as a negative control as suggested. We found that deletion of *raaR* does not affect the expression level of *gyrB* (**Fig. S3A**), and RaaR does not bind the *gyrB* promoter DNA (**Fig. S3B**). We have also evaluated the effect of one of the analogs of anthranilic acid, HHQ, on the binding between RaaR and the target promoter DNA. As shown in **Fig. S6**, we found that HHQ does not affect the RaaR binding to the target promoter DNA.

Reviewer #2: The authors should comment on the presence and conservation of RaaR within the *R. solanacearum* species complex.

Response: Thanks for your suggestion. We have added the relevant content in **Table S3** as suggested.

Reviewer #3: Major points:

1) As anthranilic acid is produced from chorismic acid and L-glutamine and this pathway is necessary to synthesis of L-tryptophan in microorganisms, it is easy to think that anthranilic acid is essential to bacterial survive in condition without tryptophan in the growth medium. Also, it seems that most bacterial species can synthesize anthranilic acid but they do not encode a RaaR homologs in their genomes (only a few groups of bacterial species that carry a raar homolog were cited in the text). In this case, most bacterial specie can synthesize anthranilic acid but may not encode for a RaaR homolog but still can communicate with other species that carries a raar homolog in their genomes (cross-talking). What the authors think about this point? Can it be better discussed in the manuscript?

Response: Thanks a lot for your good suggestion. There are two pathways identified for the synthesis of anthranilic acid. One pathway is anthranilate formation by "amide transfer," with L-glutamine as nitrogen donor. And the other is from the degradation of tryptophan via the kynurenine pathway to produce anthranilic acid. It was revealed that some bacteria present only one homolog of TrpEG or RaaR (Table S3), suggesting that some bacteria employ anthranilic acid as an intraspecies signal but other bacteria may sense anthranilic acid from hosts or microbial neighbors or only produce anthranilic acid as a common metabolic product. We have revised the discussion as suggested.

2) In material and methods are lacking description of RaaR expression in *E. coli* and purification, ITC and CD experiments including concentration of purified protein and ligand as well as instruments that were used in these studies. Also, how the QS signal molecules were isolated and quantified and the disease Index was calculated in virulence assays. Please, add more details about these experiments in Material and Methods.

Response: Thanks for your good suggestion. We have described the experimental methods in more details as suggested.

3) It will be important the authors calculate and define the interaction parameter “Kd” for the RaaR-anthranilic acid complex. This result can help in the discussion about physiological concentration of anthranilic acid in bacterial cell and in host root/stem by affecting RaaR activation.

Response: Thanks a lot for your suggestion. In chemistry and biochemistry, dissociation constant (Kd) is a specific type of equilibrium constant that measures the propensity of a larger object to separate (dissociate) reversibly into smaller components, as when a complex falls apart into its component molecules. The dissociation constant is usually denoted Kd and is the inverse of the affinity constant. The binding isotherm data showed that RaaR binds to anthranilic acid in a 1:1 stoichiometry with an estimated dissociation constant (Kd) of 3.63 μM, suggesting there is a strong binding affinity between RaaR and anthranilic acid.

4) Have the authors identified any conserved cis-element in the RaaR target promoters? If so, it will be important to generate site mutants on this putative motif to confirm the RaaR specific binding by in vitro experiments.

Response: Thanks for your suggestion. We compared and analyzed the promoter sequences of the three genes *phcB*, *solI* and *epsA*, and found that GCGGGTGCG might be the binding site. EMSA analysis showed that no DNA–protein complexes formed when the GCGGGTGCG fragment was deleted from the promoter regions of *phcB*, *solI* and *epsA* (Fig. S12), suggesting that this fragment is essential for the binding of RaaR to the *phcB*, *solI* and *epsA* promoters.

**Part III – Minor Issues: Editorial and Data Presentation Modifications**

Reviewer #1: 1. The data provided in figure S2 suggest that RaaR is regulating its own transcription. Have the authors looked into this matter?

Response: Thanks a lot for your good suggestion. In our previous ISME journal paper (Song et al., 2020. https://doi.org/10.1038/s41396-020-0682-7), the RNA-Seq result showed that the expression level of *raaR* was significant decrease in the ΔtrpEG mutant strain. And we also found that exogenous addition of anthranilic acid in the ΔtrpEG strain could restore the expression level of *raaR*, suggesting that RaaR is self-regulated.

2. It will be nice if the virulence test conducted in Fig 6 will be supplemented with representative pictures.

Response: Thanks a lot for your good suggestion. We have added the pictures of infected and control plants in Figure 6A as suggested.

3. Have the authors tested the expression or promoter activity of *epsA* in the *raaR* mutant? If not, what is the point of doing EMSA?

Response: Thanks for your suggestion. We have added the promoter activity of *epsA* in the *raaR* mutant in **Fig. S11A** as suggested.

4. As far as I know PLOS pathogens don’t have word limitation. Therefore, it is unclear to me why the materials and methods section was split between the main text and the supplementary material.

Response: Thanks a lot for your good suggestion. We have revised it as suggested.

Reviewer #2: -Line 65 – There are two more recent studies of *phc* regulation that may be worth citing here: Perrier et al. (2018), Microbial Pathogenesis (doi: 10.1016/j.micpath.2018.01.028) and Khokhani et al. (2017), mBio (doi: 10.1128/mBio.00895-17)

Response: Thanks for your good suggestion. We have added them as suggested.

-Line 273 – The authors should clarify what they mean by “fermentation broth”. If it is not CPG or a water suspension, the authors should specify the components of fermentation broth

Response: Thanks for your suggestion. We have revised it as CPG liquid medium.

-Line 426 – Did the authors perform any statistical tests on the disease progress data in figure 6A? Possible tests to perform might include repeated measures ANOVA or AUDPC (see Schandry (2017), Front Plant Sci (doi: 10.3389/fpls.2017.00623))

Response: Thanks a lot for your good suggestion. We have included the ANOVA test on the disease progress data. We have revised it as suggested (**Figure 6B**).

-Supplementary Table 2 – The authors include in this table *R. solanacearum* GMI1000 and *R. pseudosolanacearum* RS 476. If the authors are using the division of the *Ralstonia solanacearum* species complex (RSSC) into three species (Prior et al. (2016), BMC Genomics (doi: 10.1186/s12864-016-2413-z)), GMI1000 should also be referred to as *R. pseudosolanacearum*, as it is also a Phylotype I strain. Alternatively, the authors should refer to RS 476 as *R. solanacearum* and include its Phylotype. The authors should also include a line in the introduction describing the RSSC and refer to GMI1000 throughout as *R. pseudosolanacearum*.

Response: Thanks a lot for your good suggestion. We have revised *R. pseudosolanacearum* RS 476 as *R. solanacearum* RS 476 in **Table S3** as suggested.

Reviewer #3: Minor points:

Line 29: RaaR regulates the same processes as anthranilic acid, and both are conserved in various bacterial species. In this sentence should be better to say that anthranilic acid is present and not conserved in bacterial species.
Response: Thanks a lot for your good suggestion. We have revised it as suggested.
