# Supplementary material for: An anthranilic acid-responsive transcriptional regulator controls the physiology and pathogenicity of Ralstonia solanacearum
Source: PLoS Pathog. 2022 May 26;18(5):e1010562. doi: 10.1371/journal.ppat.1010562 (PMC9176790; doi:10.1371/journal.ppat.1010562)
Supplement: Attachment — Submitted filename: Response to Reviewers.docx [file ppat.1010562.s021.docx]

Dear Editor,

**RE: PPATHOGENS-D-21-02479R1**

We thank you and the reviewers for the valuable comments and suggestions again, which have been followed in the revision of our manuscript “**An anthranilic acid-responsive transcriptional regulator controls the physiology and pathogenicity of *Ralstonia solanacearum***”. As suggested, we have added one more experiment to further confirm the specific binding between RaaR and anthranilic acid (**S12 Fig**). You will find the detailed point-to-point response below. We hope that you will find our revised manuscript in this version suitable for publication in the *PLOS Pathogens*.

Sincerely yours,

Yinyue Deng

**Point-to-point response to reviewers’ suggestions**

**Part I - Summary**

Reviewer #1: The authors have successfully addressed all the issues I raised in the first review cycle. The manuscript is well written and the data is solid and presented in a clear and organized manner. I suggest some minor text revisions that will improve the paper. Outside of that, I do not have any additional requests.

Response: Thanks a lot for your comments.

Reviewer #3: The authors have answered the reviewer´s concerns and added new data which reinforce their findings. The authors described changes in RaaR secondary structure caused by anthranilic acid. Although, I think the binding of anthranilic acid and RaaR should be better explored by using some mutant in critical residues of RaaR that are involved in this interaction. The lost of this interaction should be observed in ITC experiments.

Response: Thanks a lot for your good suggestion. A previous study (Ilangovan et al., 2013. https://doi.org/10.1371/journal.ppat.1003508) showed that 9 site-specific substitution mutations in I149, A168, I186, Q194, L207, F221, Y258, I236 and I263 affected MvfR activity. To identify the critical residues in RaaR, we compared RaaR with MvfR using amino acid sequence alignments. Analysis of the two proteins identified four conserved amino acid residues at positions (A171, L191, L192 and I249) might be critical for the activity of RaaR. To investigate whether these amino acid residues of RaaR are essential for the interacting with anthranilic acid, we introduced four single site mutations in RaaR and performed ITC analysis. The results showed that the site mutations of L191 and L192 led to the lost of binding between RaaR and anthranilic acid (S12 Fig), suggesting that the L191 and L192 amino acids play an important role in the specific interaction between RaaR and anthranilic acid.

**Part II – Major Issues: Key Experiments Required for Acceptance**

Reviewer #1: None

Reviewer #3: Identification of the RaaR critical residues involved in interaction with anthranilic acid should be accomplished to give the reader a complete understanding of this mechanism. Mutant in critical residues of RaaR generated by site direct mutagenesis may help to answer this important question.

Response: Thanks for your suggestion. We have added it as suggested (S12 Fig).

**Part III – Minor Issues: Editorial and Data Presentation Modifications**

Reviewer #1: Suggested minor text modifications (lines numbers in "track changes" draft):

1. promoter fusion assays: throughout the manuscript, the authors used the term "gene expression" to describe their promoter fusions assays. In most cases, the term "gene expression" is referred to RNA accumulation (usually by RT-qPCR) and not reporter fusions analyses. The authors should clearly state in the text they used promoter activity assays to quantify gene expression.

Response: Thanks a lot for your good suggestion. We have revised it throughout the text as suggested.

2. Line 118: a sentence should not start with "and".

Response: Thanks a lot for your good suggestion. We have revised it as suggested.

3. Line 203, 205,207, and 209: trpEG and raaR should be italicized.

Response: Thanks for your suggestion. We have revised it as suggested.

4. Line 228: all the mentioned bacterial genera belong to the Burkholderiales order. This should be stated in the text.

Response: Thanks a lot for your good suggestion. We have revised it as suggested.

5. lines 260-265/Fig S13- The data presented here is more suited to be part of the result section and not the discussion section

Response: Thanks for your good suggestion. We have revised it as suggested.

6. line 294-303 (Protein Expression and Purification) - MvfR was cloned to an expression vector and the protein was purified as well. This should be added to the M&M section.

Response: Thanks for your suggestion. We have revised it as suggested.

7. line 325 – citation is missing after "as described previously".

Response: Thanks a lot for your good suggestion. We have revised it as suggested.

8. Figure 1: the title "Overexpression of the trpEG mutant with raaR" is not clear. The authors should consider rephrasing it.

Response: Thanks a lot for your good suggestion. We have revised it as suggested.

9. Figure 1A: " Effects of the homologs…". Does not describe the presented experiment well. The authors should mention that the regulators are overexpressed and that the bacteria were assayed for plate motility.

Response: Thanks a lot for your good suggestion. We have revised it as suggested.

10. Figures 3A and B/S3A/S11A: the authors should state that experiments present promoter activity analyses.

Response: Thanks a lot for your good suggestion. We have revised it as suggested.

11. Figure 6A: the authors should state in the figure legend at which time after inoculation these pictures were taken.

Response: Thanks a lot for your good suggestion. We have revised it as suggested.

12. Figure S7: ∆raaR should be italicized

Response: Thanks a lot for your suggestion. We have revised it as suggested.

Reviewer #3: (No Response)
